# Using the COVID-19 Pandemic to Assess the Influence of News Affect on Online Mental Health-Related Search Behavior Across the United States: Integrated Sentiment Analysis and the Circumplex Model of Affect

**DOI:** 10.2196/32731

**Published:** 2022-01-27

**Authors:** Damien Lekkas, Joseph A Gyorda, George D Price, Zoe Wortzman, Nicholas C Jacobson

**Affiliations:** 1 Center for Technology and Behavioral Health Geisel School of Medicine Dartmouth College Lebanon, NH United States; 2 Quantitative Biomedical Sciences Program Dartmouth College Hanover, NH United States; 3 Department of Biomedical Data Science Geisel School of Medicine Dartmouth College Lebanon, NH United States; 4 Department of Psychiatry Geisel School of Medicine Dartmouth College Hanover, NH United States

**Keywords:** affect, sentiment, circumplex, news, mental health, online search behavior, generalized mixed models, natural language processing, anxiety, depression, coronavirus, internet, information seeking, behavior, online health information, COVID-19

## Abstract

**Background:**

The digital era has ushered in an unprecedented volume of readily accessible information, including news coverage of current events. Research has shown that the sentiment of news articles can evoke emotional responses from readers on a daily basis with specific evidence for increased anxiety and depression in response to coverage of the recent COVID-19 pandemic. Given the primacy and relevance of such information exposure, its daily impact on the mental health of the general population within this modality warrants further nuanced investigation.

**Objective:**

Using the COVID-19 pandemic as a subject-specific example, this work aimed to profile and examine associations between the dynamics of semantic affect in online local news headlines and same-day online mental health term search behavior over time across the United States.

**Methods:**

Using COVID-19–related news headlines from a database of online news stories in conjunction with mental health–related online search data from Google Trends, this paper first explored the statistical and qualitative affective properties of state-specific COVID-19 news coverage across the United States from January 23, 2020, to October 22, 2020. The resultant operationalizations and findings from the joint application of dictionary-based sentiment analysis and the circumplex theory of affect informed the construction of subsequent hypothesis-driven mixed effects models. Daily state-specific counts of mental health search queries were regressed on circumplex-derived features of semantic affect, time, and state (as a random effect) to model the associations between the dynamics of news affect and search behavior throughout the pandemic. Search terms were also grouped into depression symptoms, anxiety symptoms, and nonspecific depression and anxiety symptoms to model the broad impact of news coverage on mental health.

**Results:**

Exploratory efforts revealed patterns in day-to-day news headline affect variation across the first 9 months of the pandemic. In addition, circumplex mapping of the most frequently used words in state-specific headlines uncovered time-agnostic similarities and differences across the United States, including the ubiquitous use of negatively valenced and strongly arousing language. Subsequent mixed effects modeling implicated increased consistency in affective tone (Spin_VA_ β=–.207; *P*<.001) as predictive of increased depression-related search term activity, with emotional language patterns indicative of affective uncontrollability (Flux_A_ β=.221; *P*<.001) contributing generally to an increase in online mental health search term frequency.

**Conclusions:**

This study demonstrated promise in applying the circumplex model of affect to written content and provided a practical example for how circumplex theory can be integrated with sentiment analysis techniques to interrogate mental health–related associations. The findings from pandemic-specific news headlines highlighted arousal, flux, and spin as potentially significant affect-based foci for further study. Future efforts may also benefit from more expansive sentiment analysis approaches to more broadly test the practical application and theoretical capabilities of the circumplex model of affect on text-based data.

## Introduction

News coverage can have a significant impact on mental health. In particular, the sentiment of new articles, or their views and attitudes toward specific topics, can evoke emotional responses in consumers [1-7]. For instance, news articles with a negative sentiment can elicit negative emotions on a daily basis [1], and, similarly, news articles with a positive sentiment can elicit positive emotions and enjoyment in consumers [2]. The intensity of the emotional response to the news is strongly related to the personal relevance of stories [1,3]. Emotional intensity is also contingent on the type of news: forms of “hard” news such as significant world events and more pressing issues (eg, natural disasters, political turmoil) typically evoke stronger responses than “soft” news articles that are more sensational and less timely (eg, sports, pop culture, tabloid-style stories) [2,4]. Furthermore, online sources such as social media are being increasingly used as a source of news [8], and posts seen in news feeds have shown to elicit emotional responses aligning with the sentiment of the posts [5]. Given the impact of daily emotional responses on long-term mental health (eg, [9]), it is worth investigating the role of daily stressors such as news coverage sentiment in the mental health of consumers.

News headlines are a specific facet of news coverage that can impact mental health. Headlines attempt to draw attention to specific details and facts about the main story, often significantly impacting how the reader perceives the given information [6]. To attract the reader, news headlines will often contain strong sentiment [6,10], leading readers to experience emotional responses from simply reading headlines [7]. Furthermore, social media outlets such as Facebook are being increasingly used as news sources, and estimates suggest that over 90% of Facebook users only read the headlines of news stories in their feeds [11]. Thus, while both story headlines and content are important to consider, specific focus on headlines may be more prescient from a mental health perspective.

A recent global issue of pervasive personal relevance and therefore particular noteworthiness when considering the mental health implications of news coverage is the COVID-19 pandemic. Recent studies have reported that people respond adversely to news pertaining to COVID-19. One study found that over one-third of participants were spending at least two hours on social media reading COVID-19–related news, and extended exposure to COVID-19 news was associated with higher anxiety and depression in adults [12]. More extreme exposure (eg, ≥3 hours) has been found to be associated with generalized anxiety disorder [13]. Studies have led to similar findings among college-aged students, reporting that students spending an hour or more online looking for COVID-19–related news have significantly higher levels of anxiety and somatization [14]. In a separate multimodal passive sensing study with self-reported ecological momentary assessments conducted on 217 undergraduate students in the United States, students were found to be more sedentary and reported increased anxiety and depression symptoms (*P*<.001) during the academic term coinciding with the first few months of the COVID-19 pandemic (Winter 2020) compared with previous academic terms [15]. Additionally, movement and sleep-related behaviors were found to be associated with fluctuations in COVID-19 reporting [15]. Furthermore, another study looked specifically at the sentiment of headlines in COVID-19 news articles and found that over 50% of headlines had a negative sentiment, 30% had a positive sentiment, and under 20% had a neutral or nonpolarizing sentiment [16]. Taken together, there is empirical precedent to link the consumption of COVID-19–related news to changes in mental health.

Given that the majority of people under 25 years perform internet searches as their primary method of seeking mental health information and help [17], it stands to reason that internet search behavior may be a novel and powerful indicator of mental health changes. With readily available data accessible via platforms such as Google Trends, internet search behavior represents a large corpus of data with increasing prevalence in epidemiological applications. For example, peaks in suicide search term activity correspond to high-profile news stories pertaining to suicide [18] as well as completed suicide rates in many countries [19]. Along with this, internet searches exhibit seasonal variability, with peaks in mental health searches occurring in the winter and troughs occurring in the summer, aligning with seasonal depressive disorder [20]. Furthermore, mental health searches in the United States spiked in early 2020 after lockdown announcements due to the COVID-19 pandemic, but such searches leveled off in response to the issuing of stay-at-home orders [21]. All in all, the leverage of records derived from internet search behavior represents a broader method of sampling that may have implications for population-level mental health—a degree of heterogeneity that is not always attainable through more traditional epidemiological or clinical sampling approaches.

Given the versatility of internet search trends as an indicator of mental health changes, a popular and successful approach to analyzing such text corpora is using natural language processing (NLP). NLP is a computational-based approach to analyzing text, and it has been used to study and model a variety of mental health constructs [22-27]. A particularly useful facet of NLP is sentiment analysis, which is a method of computationally assessing opinions, subjectivity, and emotion in text [22]. At a basic level, sentiment analysis constitutes the extraction of words and phrases (n-grams) from a text corpus to compare these features with prior knowledge regarding their affective connotation. A common approach to extraction is to use a rule-based technique, which applies a consistent system of manually derived rules for reference and identification of sentiment, such as a dictionary mapping of words to a specific sentiment category or value (eg, positive or negative) [23]. Studies leveraging sentiment analysis have also relied on automatic techniques that operate by implementing machine learning–based methods, which classify the sentiment of novel text by “learning” from the features of example data (eg, [24]). In more recent literature, hybrid techniques combining elements of both rule-based and automatic techniques have emerged, often being used for analyzing sentiment across domains (eg, [25,26]). Sentiment analysis has been commonly applied to text from social media platforms such as Twitter. For instance, Chakraborty et al [27] analyzed the sentiment of tweets to assess mental health in response to COVID-19, and Chintalapudi et al [28] applied a hybrid-based sentiment analysis using a naïve Bayes classifier to patient medical records to assess trends in patients’ physical and mental health over time. In the broadest sense, this methodology has presented as a promising means to quantitatively ascertain mental health dynamics.

The circumplex model of affect is a theory-driven approach to ascertain emotional dynamics from the quantitative perspective of sentiment analysis. One version of the model posits that, for each individual, all affective states stem from 3 neurophysiological states: valence (pleasure/displeasure), arousal (alertness/excitation), and dominance (autonomy/restriction) [29]. Specifically, all emotions can be represented as a linear combination of valence, arousal, and dominance, or different degrees of these states [29]. It is worth noting that affect, sentiment, and emotion, while they are often regarded as equivalent and used interchangeably, are distinct in the psychological literature. Affect is considered an “umbrella term” that encompasses both emotion and sentiment which refers to an individual’s subjective response to an experience, which can vary in positive and negative intensity [30]. Emotion is a conscious affective experience that is an adaptive response to some event which, according to circumplex theory, can be measured by its valence and arousal [30]. Finally, sentiment is an individual’s acquired affective disposition toward an object or event, wherein a disposition will have emotions associated with it [30]. Thus, the affect circumplex inherently refers to both sentiment and emotion. Preceding the valence–arousal–dominance (VAD-3D) circumplex model is the valence–arousal (VA-2D) circumplex model [31]. While the VA-2D model has the earliest and widest empirical support in the literature (eg, [31,32]), the VAD-3D model has shown to be more sensitive in the differentiation of certain affective states (eg, fear and anger) compared with the VA-2D model (eg, [29,33]). In general, a circumplex model can be constructed for an individual by assessing affective states after multiple interactions over time. Further analysis of the circumplex dimensions offer deeper understanding of an individual’s emotions and emotion dynamics, or how an individual’s emotions change over time.

Although the affect circumplex has traditionally been used to temporally model emotion in individuals, it has also been used to operationalize word usage within the context of sentiment analysis. For instance, the circumplex model has been applied to detect sentiment from social media content, including Twitter tweets [34] and Facebook posts [35]. While there have been several disparate and effective empirical applications of the affect circumplex to the greater sentiment analysis framework, the properties of the circumplex itself have also been utilized within the research domain of emotion dynamics. In a meta-analysis, researchers examined the flux, pulse, and spin of affect [36] to predict changes in aggressive behaviors and found that flux in positive affect, contrary to expectation, was associated with individual aggression [37]. More broadly, their findings supported the application of such dynamic measures to better understand human social behavior [37]. While currently underrepresented in the literature as a suite of metrics within the NLP toolkit, there is broader empirical precedent to apply the affect circumplex as a uniquely synergistic operationalization of emotion/sentiment. Inspired by the promise of circumplex theory and application within the extant literature, this study aimed to integrate notions of circumplex dynamics with a medium of the written word, thereby leveraging novel quantifications of semantics for exploratory and hypothesis-testing applications within the mental health space.

The COVID-19 pandemic, as of June 2021, has claimed the lives of nearly 4 million individuals worldwide [38], significantly altering the infrastructure, enterprise, and sociobehavioral landscape of the globe. Its widespread, devastating impact to physical, mental, and economic well-being has presented a unique case study opportunity to further explore how news media coverage of such a ubiquitous, baleful issue influences the mental health of a society that is now highly integrated with, and reliant on, digital technology for information exchange. The breadth and depth of the data associated with analyses on this front necessitate creative and novel analytical approaches to uncover nuanced patterns that may have potential implications for how information is expressed as well as for the well-being of those who are exposed to its expression. Given the broadly unifying and consistently impactful nature of the recent pandemic, the current study sought to interrogate the association between the affective dynamics in digitally accessible local news media and online trends in mental health search behavior across the United States. This work capitalized on the affordances of online “big data” and leveraged both Google Trends and Media Cloud repositories to collect daily, state-specific, concurrent information on internet search activity and written news media coverage of the pandemic, respectively. Data from March 24, 2020, to October 22, 2020, were used to specifically profile COVID-19–related news article headlines based on affect. As mentioned in previous work, a focus on news headlines provides several benefits: (1) they are dense with contextual information while being short and more consistently formatted, (2) they often appeal to readers’ emotions, and (3) avoid issues of data privacy [7]. Relying on NLP techniques to ultimately draw from the circumplex theory of affect and associated operationalizations of emotion dynamics, in conjunction with generalized mixed statistical modeling to assess online mental health behavioral correlations with these operationalizations, this research contributes a novel, integrative analytical framework for the exploration and quantification of news media affect in the digital era.

To this end, the current body of work consisted of a descriptive/exploratory aim leveraged to inform subsequent research questions aimed to model online mental health–based search behavior in a targeted and empirically justified manner. Accordingly, the first aim was to gain some intuition regarding the language surrounding written COVID-19 news reporting and characterize the semantic affect of written COVID-19 news reporting across the United States. This aim was expressed in terms of 2 initial exploratory research questions:

1. How does affect (valence, arousal, and dominance) of state-specific COVID-19–related news headlines change throughout the course of the initial phases of the pandemic?
2. How different is overall word usage in COVID-19–related news headlines across the country?

The statistical and qualitative answers to these questions at baseline were then used to inform and justify subsequent modeling decisions for the second aim, namely, to interrogate the association between mental health–based online search behavior and the affective dynamics of COVID-19 news exposure through time. Specifically, the resulting models leveraged operationalized word affect exposure and focused on the potential utility of affective dynamics in circumplex space to model changes in online mental health search behavior through time. As a result, this second aim was driven by the following 2 additional research questions:

1. Do variables that reflect dimensional circumplex dynamics of affect (flux, pulse, and spin) across daily news story headlines have a stronger, statistically significant association with same-day online mental health search behavior outcomes compared with more simple measures of affect (ie, daily average valence, arousal, and dominance)?
2. Given the core importance of valence and arousal in affect theory, are features that capture the valence and arousal components of news headlines more significant and more highly associated with mental health search behavior outcomes compared with dominance-related metrics?

## Methods

### Study Sample

This work used publicly available COVID-19–related online news headlines. As described in more detail in the “News Data” section, 88,987 news story titles were analyzed in total and represented written coverage of the pandemic through time across the top-ranked state-based news outlets by web traffic in the United States (n=135 news outlets).

### Data Collection

#### News Data

News outlets were selected to represent daily sources that have the largest audience while also emphasizing coverage of local state-wide news, thus broader news outlets with substantial national or international coverage were not selected (eg, the New York Times, the Washington Post, USA Today). Web traffic was used as a heuristic to select representative news outlets on a per-state basis, with the requirement that the source ranked within the top 5 of all outlets for that state [39]. Sources were not considered if they were not among the most frequently visited. In many cases, sources that ranked highly in terms of web traffic were also ranked within the top 10 in terms of physical circulation [40] (Multimedia Appendix 1). As the study aimed to investigate the association between online search behavior (operationalized through Google Trends data) and the language of news coverage, it was most practical to focus on news information readily available via the web. To this end, the study utilized the Media Cloud API client, an open-source platform for tracking millions of stories published online, to ultimately construct a web scraping pipeline in Python and programmatically extract the titles, dates, and body text from stories of selected news sources [41,42]. COVID-19–related news stories were queried from January 23, 2020, to October 22, 2020, for the presence of “covid”, “covid19”, “covid-19”, “coronavirus”, or “pandemic” in at least one of either the story headline or main body text.

The study aimed for 3 news sources per state, but due to the limitations of data availability via Media Cloud as well as paywalls specific to certain news outlets, several states did not have appreciable representation of stories from top-ranked outlets available for access between the dates specified for the study. Typically, if Media Cloud had insufficient data for a news source (defined as <15 stories per month on average), the news source with the next highest web traffic rate was chosen (up to the fifth highest ranked news source). An exhaustive list of the sources used and their respective Media Cloud identifiers is provided in Multimedia Appendix 1.

The analyses for this work were conducted specifically on the content of story headlines. All stories were manually curated to remove advertisements and other non-news content as well as duplicate entries within any 1 news source. Stories without an associated date of publication were removed. In several cases, body text was not accessible; however, the headlines of these stories were still included for analysis. The numbers reported in Multimedia Appendix 1 represent the outlet-specific totals for analysis after curation and preprocessing. Despite the availability of news data from January 23, 2020, only data starting from March 24, 2020, to October 22, 2020, were used for downstream hypothesis testing, as the initiation of data collection associated with the outcomes of interest (see the “Mental Health Search Activity” section) occurred on March 24, 2020.

#### Mental Health Search Activity

Counts of mental health–related search terms were collected daily using the *gtrendsR* (v1.4.8) package in the R programming language. For each of 17 mental health terms (“anxiety,” “depression,” “hopeless,” “angry,” “afraid,” “apathy,” “worthless,” “worried,” “restless,” “irritable,” “tense,” “scattered,” “tired,” “avoiding,” “insomnia,” “suicidal”, and “suicide”), queries were conducted for search activity across the United States with state-level resolution. Following the authors’ previous work in this domain [21], these terms were adapted and validated in part by prior research on mental health using Google Trends [20] as well as by research that assessed rapid affective symptom changes in accordance with the Diagnostic and Statistical Manual of Mental Disorders, 5th edition [43,44].

As discussed in more detail in the Google Trends documentation, search data are automatically normalized and scaled from 0 to 100 based on a topic’s proportion to all searches on all topics within a specified period and geographic location. However, for this work, raw counts of searches were desired rather than normalized values. To ultimately arrive at estimated raw counts of mental health term searches, queries in *gtrendsR* were constructed to include a unique daily comparator term that was selected to serve as a representative top-trending term for that day. These trending terms are published daily by Google in tiers of absolute frequency (eg, >50,000 hits) and are therefore not normalized. Each programmatic call to *gtrendsR* therefore consisted of 2 simultaneous keywords (eg, “anxiety” and “NASA”) corresponding to a mental health term and the selected comparator term for that day. Manual selection of comparator terms was performed to ensure that the term was as unrelated as possible with regard to both COVID-19 and mental health. The complete list of utilized comparator terms by date, along with their estimated search volume thresholds, is provided in Multimedia Appendix 2. Ultimately, each query to *gtrendsR* returned state-level normalized counts for the target mental health term and the representative comparator term for the date of interest. From this information, a state-specific estimated daily count for each mental health term was calculated in the following manner:

MH_NormTot_ × (COMP_SV_)/(COMP_NormTot_) = MH_AdjTot_ (**1**)

MH_AdjTot_ × (MH_NormState_)/(MH_NormTot_) × (POP_State_)/(POP_Tot_) = MH_AdjState_ (**2**)

In Equation 1, an adjusted count for the target mental health term MH_AdjTot_ was first calculated by scaling the *gtrendsR* normalized total count of the target mental health term (MH_NormTot_) by the ratio of the reported comparator term search volume to its *gtrendsR* normalized total count (COMP_SV_/COMP_NormTot_). Because Google reports search volume in tiers (eg, 50,000+, 100,000+), the value of COMP_SV_ was selected to be the lowest possible value that represents that tier (eg, an estimated search volume of 50,000+ was set to correspond to a COMP_SV_=50,000). MH_AdjTot_ is summative across all 50 states. To distill a practical estimation of mental health search term counts at the state level, Equation 2 then takes the ratio of the normalized target state count to the normalized total count (across all 50 states) for the target mental health term (MH_NormState_/MH_NormTot_) as well as the estimated ratio of the population belonging to the target state (POP_State_/POP_Tot_) (calculated from information provided by the 2019 US Census Bureau) and computes the product of these values to ultimately calculate the target state–specific portion of MH_AdjTot_ and yield MH_AdjState_. Resulting counts were rounded down to the nearest whole number. The process was repeated for each of the 17 mental health terms, for each of the 50 states, across the 213 days of the study spanning from March 24, 2020, to October 22, 2020. Quantification and preprocessing of raw *gtrendsR* count data were performed with custom scripts written in the Python programming language (version 3.8.3).

### Sentiment Analysis

Quantification of the emotional tone of words is a major analytical arm of NLP. A component and feature of many NLP analytical suites such as Python’s *nltk* library [45], sentiment analysis commonly involves quantification of both polarity (valence) and intensity (arousal) of emotion. While it is common to employ pretrained models or dictionaries with packaged implementations such as what is provided through *nltk*, it is also possible to conduct sentiment analysis with a custom reference dictionary so as to capture additional or unique nuances of emotion. Accordingly, this work leveraged a large, comprehensive, and empirically derived reference dictionary of 13,915 English lemmas quantified by a crowdsourcing effort involving 1827 responders and over 1 million word ratings of valence, arousal, and dominance [46]. Average (SD) valence of all rated words in the dictionary is 5.064 (1.275), average arousal is 4.211 (0.986), and average dominance is 5.185 (0.938) on a scale of 1-9, with higher values indicative of more positive polarity, stronger intensity of emotion, or more dominant language. For this study, all individual word ratings of valence, arousal, and dominance were standardized to obtain Z-score equivalents prior to application in downstream analyses. As a result, numerical values represent SDs below or above the average (Z=0) across all reference dictionary words for that affective dimension.

With a custom-built Python script, tabular data containing a state’s news story titles and dates of publication were first preprocessed using *nltk* and regular expressions to lowercase all words, remove all nonalphabetical characters, and remove common stop words—words that do not add meaning or provide context to a sentence (eg, “the,” “she,” “it”). Additional words were manually appended to the default stop word dictionary to account for location names (eg, “orange,” “lake,” “phoenix”), media language (eg, “video,” “subscribe,” “share”), and proper nouns doubling as reference dictionary entries (eg, “mitt,” “trump”). A complete list of manually added stop words is available in Multimedia Appendix 3 for reference. News stories were then grouped by date of publication, and all words across story titles were pooled into date-based lists representing a given state’s headline word usage across time. From these lists, the average Z-score valence, arousal, and dominance across words that mapped to the reference dictionary were calculated per day. This process was repeated for each of the 50 states to arrive at state-specific, daily averages of normalized news headline sentiment across 3 primary affect dimensions. Out of the total 731,607 non–stop words across all news story titles in the data set, 252,524 (34.52%) mapped to the reference dictionary and were therefore considered in analyses.

### Descriptive Statistics of State-Specific Affect Through Time

To address the exploratory component of this study, several approaches were taken. With regard to the first question of affect across time, the news collection period (January 23, 2020-October 22, 2020) was first divided into 5 distinct temporal windows comprising 3 phases of the pandemic in the United States. The first phase is referred to as “Pre-pandemic” and spans 50 days (January 23, 2020-March 12, 2020). The termination of this phase and the initiation of the second phase was defined by the official presidential announcement (Proclamation 9994) defining COVID-19 as a national emergency on March 13, 2020. The second phase, “Early Response,” spans 26 days and terminates on April 7, 2020. This date marks South Carolina’s announcement of stay-at-home orders, the last of 42 states to make an official statement regarding the required alterations to daily behavior and movement. The third and final phase, “Mid-pandemic,” continues for 198 days to the end of the study period (April 8, 2020-October 22, 2020). This phase was further subdivided into 3 equal partitions of 66 days to create event-agnostic temporal parity *within* the phase. This also allowed for less biased statistical comparisons *across* all 3 phases because the resulting statistics summarized phases with more equivalent data representation.

News headlines were then parsed based on publication date into these 5 temporal bins. For each state, the (1) mean, (2) variance, and (3) root mean square of successive differences (RMSSDs) [47] for the standardized daily averaged valence, arousal, and dominance were calculated. Where mean and variance calculations represented more traditional summative statistical parameters and defined a phase more broadly, RMSSD captured notions of change and consistency in affect within temporal partitions over time. With the resulting data in hand (see Multimedia Appendix 4 for each state’s partition-specific data), affect-specific choropleths for each of the 5 temporal windows were constructed. In addition, 6 representative states were selected (Arizona, Georgia, Illinois, New Jersey, Texas, and Washington) to further illustrate similarities and differences in state-specific affect mean and RMSSD across the designated phases of the pandemic. These states were chosen for further illustration based on their collective geopolitical breadth as well as their appreciable volume of available news headlines; however, all states were analyzed in subsequent modeling efforts (see the “Generalized Mixed Effects Modeling” section). The results of these initial collective efforts served in part as the impetus for further pattern-based operationalizations (see the “Affect Circumplex Feature Engineering and Exploratory Visualization “ section) and hypothesis testing (see the “Generalized Mixed Effects Modeling” section).

### Affect Circumplex Feature Engineering and Exploratory Visualization

#### Overview

Appreciable patterns and variability in affect RMSSD across time compared with mean and variance (see the “Descriptive Statistics of State-Specific Affect Through Time” section) suggested promise in leveraging signatures that capture the dynamics of affect expression to ultimately integrate the circumplex model of affect with sentiment analysis. Where observed differences in RMSSD across states argued for potential utility in modeling day-to-day volatility of affect, observed differences in RMSSD across time within any 1 state argued for potential utility in modeling the course of this affect volatility. The circumplex, while not interrogating volatility per se, nevertheless provides a practical, theory-guided means with which *patterns* in written sentiment can be quantified and further investigated within a temporal modeling framework. As such, several additional features were derived from the daily normalized average values of valence (*V*), arousal (*A*), and dominance (*D*) for each state’s news headlines calculated in the “Sentiment Analysis” section. These features served as key variables in subsequent modeling efforts (see the “Generalized Mixed Effects Modeling” section) to uniquely operationalize affective variability in VA-2D and VAD-3D circumplex space and ultimately interrogate the significance of daily patterns in sentiment expression as associated with online mental health search behavior outcomes (see the “Mental Health Search Activity” section).

#### Flux

An operationalization of affect consistency along a dimensional axis of the affect circumplex, flux is the SD of average Z-standardized word affect scores (Flux_V_, Flux_D_, Flux_A_) across all mapped words pooled from all state-specific news story headlines for the day. Flux is scalar—it focuses on intensity and is agnostic to directionality of the word vector in circumplex coordinate space.



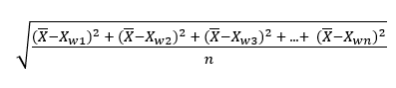
 (**3**)


where 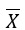 is the mean standardized affect scores across all words, *w*_1_, *w*_2_, *w*_3_, ..., *w*_n_.

#### Pulse

An operationalization of the consistency in extremity (distance from the origin), pulse is the SD of the word vector magnitudes in 2D circumplex space (Pulse_VA_, Pulse_VD_, Pulse_AD_)*.* Pulse is similar in mathematical form (an SD) to flux, except that the SD is found on a set of vector magnitudes rather than scalar quantities of affect—unlike flux, pulse considers both intensity and direction. The vector magnitude, *M*, for a word, *w*, was operationalized in the circumplex as the Euclidean distance in Cartesian coordinate space from the origin (0,0) to (*V_w_*, *A_w_*) in the VA circumplex, to (*V_w_*, *D_w_*) in the VD circumplex, and to (*A_w_*_,_ *D_w_*) in the AD circumplex.



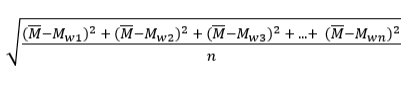
 (**4**)


where 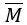 is the mean of the vector magnitudes across all words modeled in the circumplex.

#### Spin

An operationalization of the consistency in angular orientation or the breadth of coverage across the affect space (Spin_VA_, Spin_VD_, Spin_AD_), spin is the SD of the word vectors characterized in terms of their angular displacement from the horizontal 2D circumplex axis (0°). Spin is the opposite of flux in that it is only concerned with position and is therefore intensity agnostic. Like both flux and pulse, spin is at its core a calculation of an SD; however, it is the SD of vector angles (from the x-axis) rather than vector magnitudes or scalar affect values. Thus, the angle formed between the word affect vector and the x-axis of the circumplex, *θ*, for a word, *w*, is equivalent to the cosine of the dot product of vectors *u* = (*a_x_*, 0) and *v* = (*a_x_*, *a_y_*), where *a_x_* and *a_y_* are the x-dimensional and y-dimensional affect values (*V*, *A*, or *D*) associated with the 2D circumplex of interest, respectively, divided by the product of their magnitudes:

cosθ*_w_* = (*u*·*v*)/||(*u*·*v*)|| (**5**)

The final SD equation for calculating spin, with 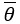 as the mean of the angular displacement calculations across all words modeled in the circumplex, is therefore:



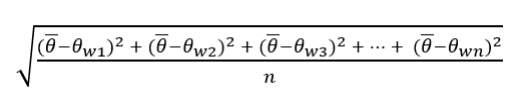
 (**6**)


**Figure 1:**
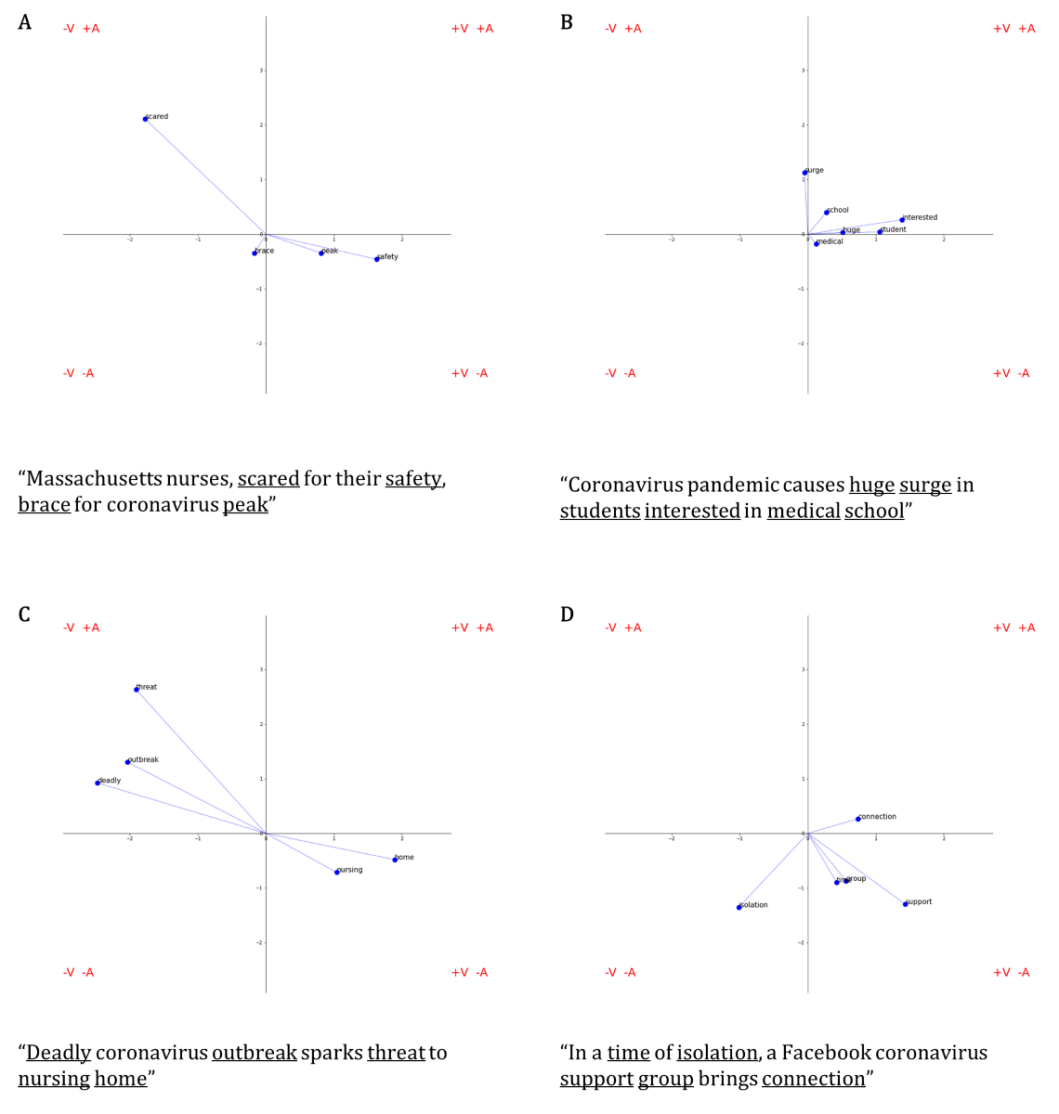
Example valence-arousal affect circumplex plots for COVID news headlines. Headline words that map to the affect dictionary are plotted in affect space with the x-axis representative of a word’s valence and the y-axis representative of a word’s arousal. The units of both axes represent the Z-score of the word; thus, they can be interpreted as standard deviations from the mean affect score across all words in the reference affect dictionary. Within this derived coordinate space, the circumplex properties of pulse and spin can be intuitively visualized through the mapping of example news headlines. (A) Higher spin and higher pulse. (B) Lower spin and higher pulse. (C) Higher spin and lower pulse. (D) Lower spin and lower pulse.

To provide summative visualizations of descriptive patterns in news headline word choice within the circumplex framework, VA-2D word clouds were derived for each of the 6 representative states from the “Descriptive Statistics of State-Specific Affect Through Time” section (Arizona, Georgia, Illinois, New Jersey, Texas, and Washington). While the efforts outlined in the aforesaid section focused on the deconstruction of basic affect parameters at distinct phases of the evolving pandemic, the efforts herein served 2 purposes: (1) visually introduce the more nuanced circumplex and (2) more broadly characterize word usage among states in a time-agnostic manner. To develop some intuition, Figure 1 provides simple descriptive examples of affect flux and spin profiles from singular Massachusetts news headlines. In practice, however, the resulting circumplex plots represented the most frequently mapped words (≥2 SDs above the mean count of words across news headlines in that state) from a state-wise concatenation of all news headlines across time.

### Generalized Mixed Effects Modeling

To investigate the association between mental health search term activity and affective dynamics of COVID-19 news reporting across the United States, 8 separate mixed effects models were constructed. Because both the VA-2D and VAD-3D operationalizations of affect are popular, the 8 models represent 4 pairs of outcome-specific investigations, where each pair consists of 1 model with VA circumplex variables and another model with VAD circumplex variables (see the “Affect Circumplex Feature Engineering and Exploratory Visualization” section). For each model pair, the outcome was operationalized differently to both holistically and more specifically interrogate mental health search behavior. One subset of the selected terms most closely corresponded with the mental health construct of depression, another distinct subset corresponded with anxiety, and a third aligned with both depression and anxiety. Accordingly, the first model, “All Cluster,” pooled the daily state-level counts for all 17 mental health search terms into a single sum; the second model, “Depression Cluster,” pooled the daily state-level counts for “apathy,” “depression,” “hopeless,” “suicidal,” “suicide,” “tired,” and “worthless” into a single sum; the third model, “Anxiety Cluster,” pooled the daily state-level counts for “afraid,” “anxiety,” “avoiding,” “restless,” “tense,” and “worried” into a single sum; and the fourth model, “Nonspecific Cluster,” pooled the daily state-level counts for “angry,” “insomnia,” “irritable,” and “scattered.” Given that these outcomes were overdispersed count data (ie, with the variance of the outcome distributions higher than the mean), models were constructed as negative binomial mixed models in R using the *glmmTMB* package (version 1.0.2.1) [48]:

MHClusterCount_VA_ = t + V + A + Spin_VA_ + Pulse_VA_ + Flux_V_ + Flux_A_ + (1 | State) (**7**)

MHClusterCount_VAD_ = t + V + A + D+ Spin_VA_ + Spin_VD_ + Spin_AD_ + Pulse_VA_ + Pulse_VD_ + Pulse_AD_
**+** Flux_V_ + Flux_A_ + Flux_D_ + (1 | State) (**8**)

For both the VA-2D (Equation 7) and VAD-3D (Equation 8) models, time (day count since March 24, 2020), *t*, and each of the respective affect values and circumplex features were included as fixed effects, while state was included as a random effect. All negative binomial models were run on stacked format tabular data by specifying “family=nbinom2” with default settings in *glmmTMB*. Model fit was assessed using Efron pseudo-*r*^2^ [49]*.* See Multimedia Appendix 5 for the complete data set used in analysis. For closer intuitive introspection of marginal and interaction effects, prediction plots of the most statistically significant (*P*<.001) variables were constructed using the *sjPlot* package (version 2.8.7) in R [50].

### Ethics

This paper was not considered human subjects research because it used anonymous, publicly available data and as such was exempt from human subjects approval.

## Results

### Study Sample

**Figure 2:**
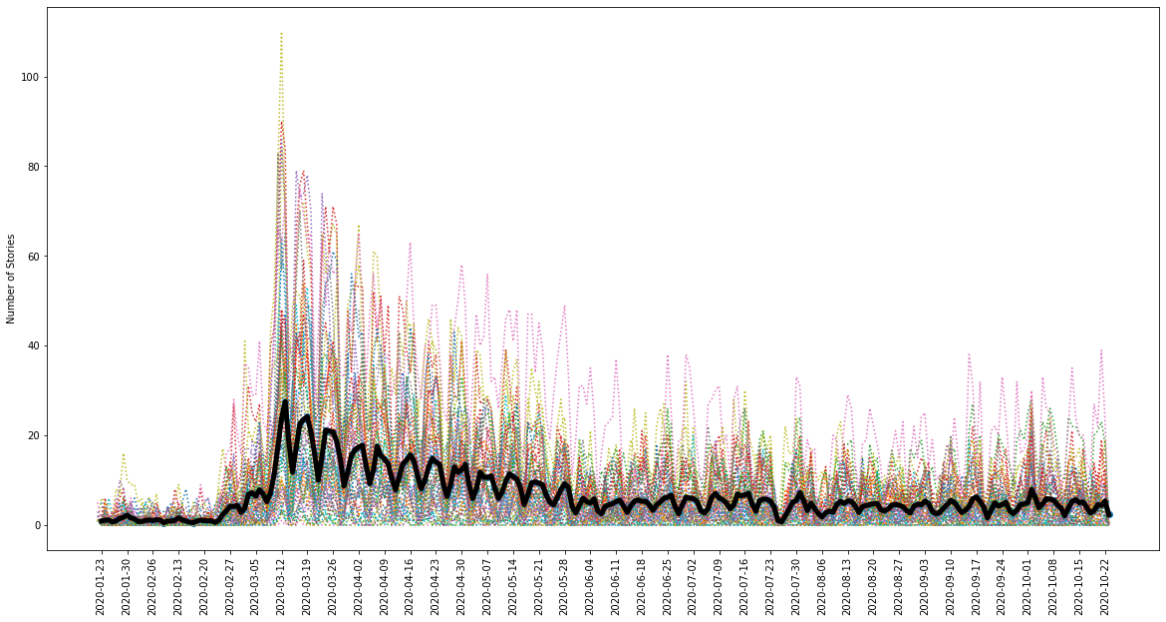
Trend in available number of COVID-19 news stories across the United States. Colored dashed lines represent state-specific trends in available COVID-19-related news stories through time as determined by the accessibility of the selected news outlets’ story headlines via Media Cloud. The bold black line indicates the trend in the average number of representative stories across states. The maximum average number of stories (27.5) occurs on March 13, 2020, coinciding with the release of the presidential Proclamation 9994 that declared COVID-19 a national emergency.

Figure 2 illustrates the patterns in overall and state-specific COVID-19 news availability in the current data set. General trends reflect the progression of the pandemic in the United States, with the highest representative coverage occurring in mid-to-late March 2020, coinciding with the official US presidential announcement of a national emergency and the first wave of state-specific announcements and enactments of stay-at-home orders that shortly followed [21]. For a complete list of the outlets utilized by state, along with their respective details on publication city, web traffic, and state circulation rankings, as well as the number of stories included in the data set, please refer to Multimedia Appendix 1.

### Descriptive Statistics of State-Specific Affect Through Time

**Figure 3:**
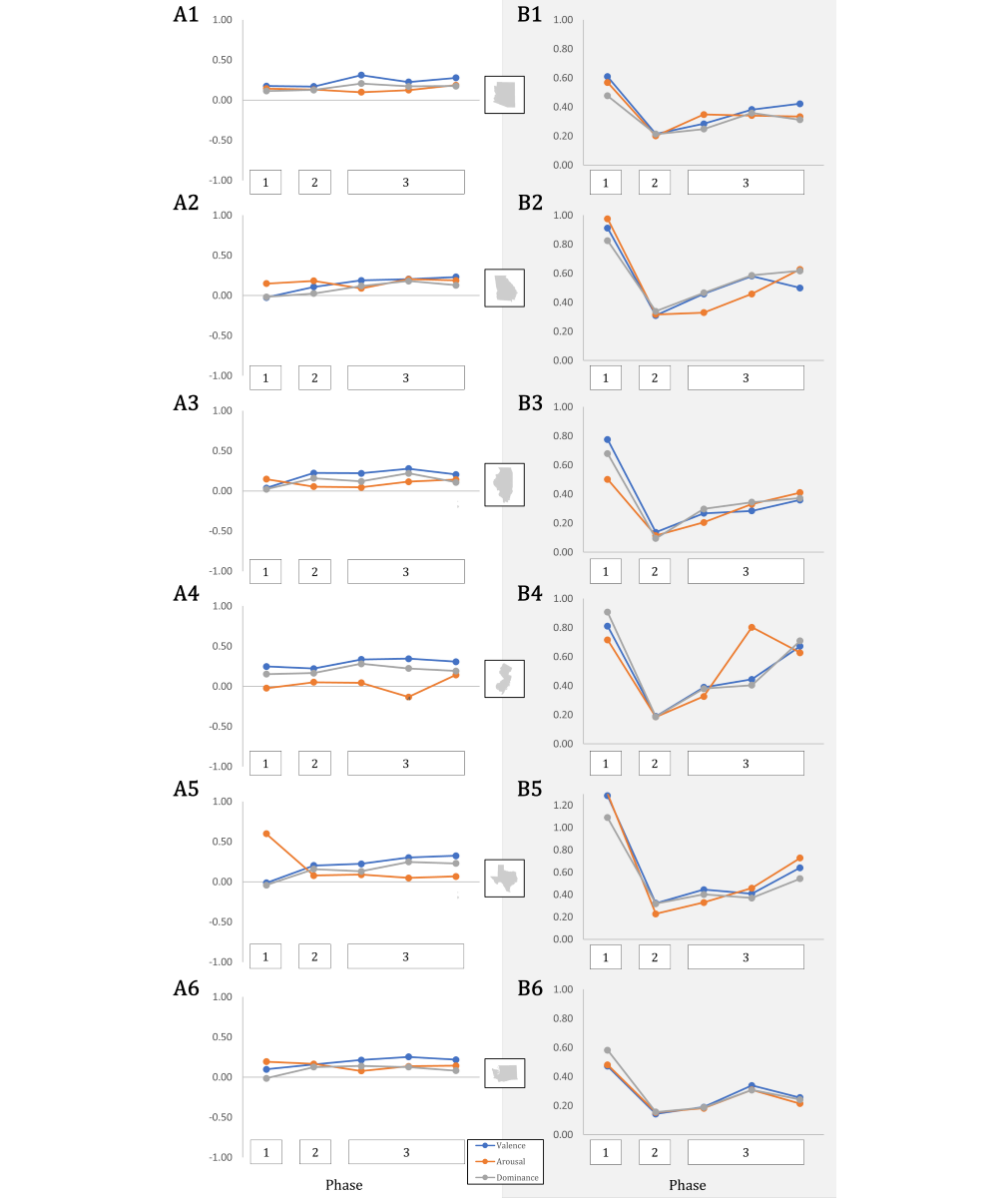
Affect trajectories of representative states across phases of the COVID-19 pandemic. The (A) mean and (B) RMSSD of phase-specific standardized valence (blue), arousal (orange), and dominance (green) across all words comprising COVID-19-related news headlines for each of (1) Arizona, (2) Georgia, (3) Illinois, (4) New Jersey, (5) Texas, and (6) Washington. RMSSD: root mean square of successive differences.

Phase-based calculations of standardized affect mean, variance, and RMSSD for each of the 50 states’ news headlines revealed several interesting features of the data. In general, the summative phase metrics of mean and variance for each of valence, arousal, and dominance did not exhibit any meaningful trends across phases. For example, all 3 measures of affect tended to hover between a mean standardized score of 0.0 and 0.5, indicating an overall propensity toward slightly positive, arousing, and dominant language in COVID-19–related news headlines in each phase. However, the RMSSD, which captured the change in day-to-day affect over time within each phase, exhibited a general pattern across phases that was consistent in a majority of states. This pattern is clearly illustrated in the 6 representative states of Figure 3B(1-6) and can be described as low consistency (higher RMSSD) in affect during Phase 1, followed by an appreciable increase in consistency (lower RMSSD) from Phase 1 to Phase 2, leading ultimately to a gradual “rubber-banding” back toward lower consistency across the 198 days of Phase 3. This pattern was observed regardless of the affect dimension in question. Discrepancies between states manifested in terms of the overall magnitude in these shifts, including the extent of “deterioration” in headline affect consistency during Phase 3, thus indicating that despite general similarities in news affect across the nation, local news sentiment over time at different phases of the pandemic is idiosyncratic to the state in question. In turn, this suggests that a fuller appreciation of affect differences in news headlines requires operationalizations that emphasize nuanced *patterns* of expression.

**Figure 4:**
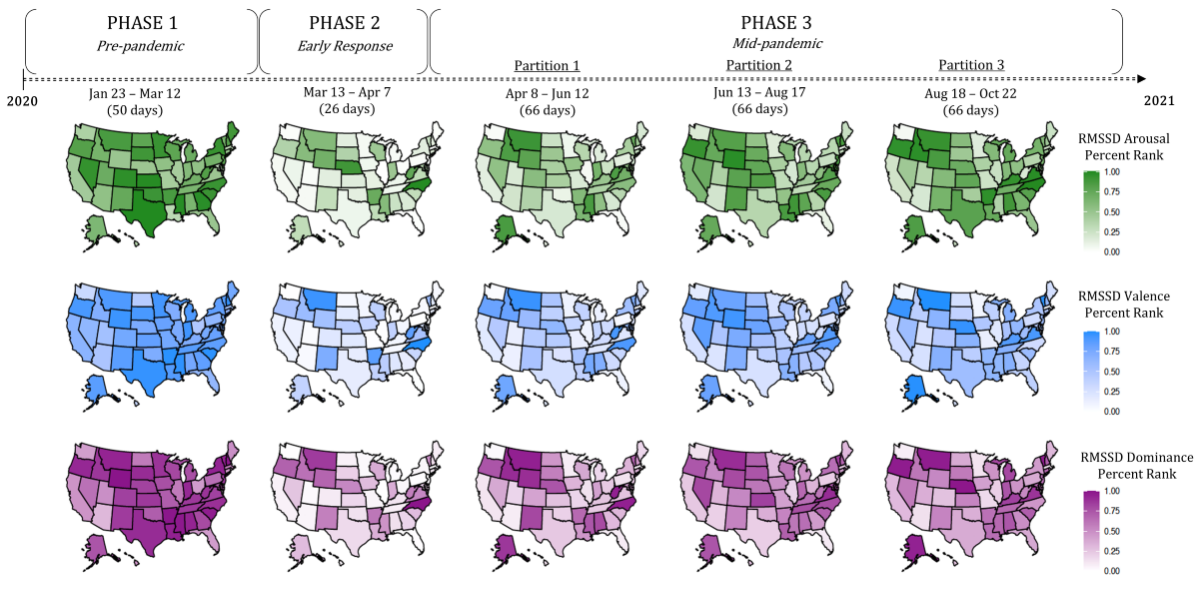
Choropleths of news affect percentile ranked RMSSD across phases of the COVID-19 pandemic. The percentile rank of state-specific arousal (green), valence (blue), and dominance (purple) RMSSD across three phases (five temporal partitions) of the pandemic. A pattern of high RMSSD during Phase 1, low RMSSD during Phase 2, and a gradual increase in RMSSD throughout Phase 3 is characteristic of the majority of states. As shown, the temporal window of the study (January 23, 2020 - October 22, 2020) represents the first nine months of the pandemic. RMSSD: root mean square of successive differences.

**Figure 5:**
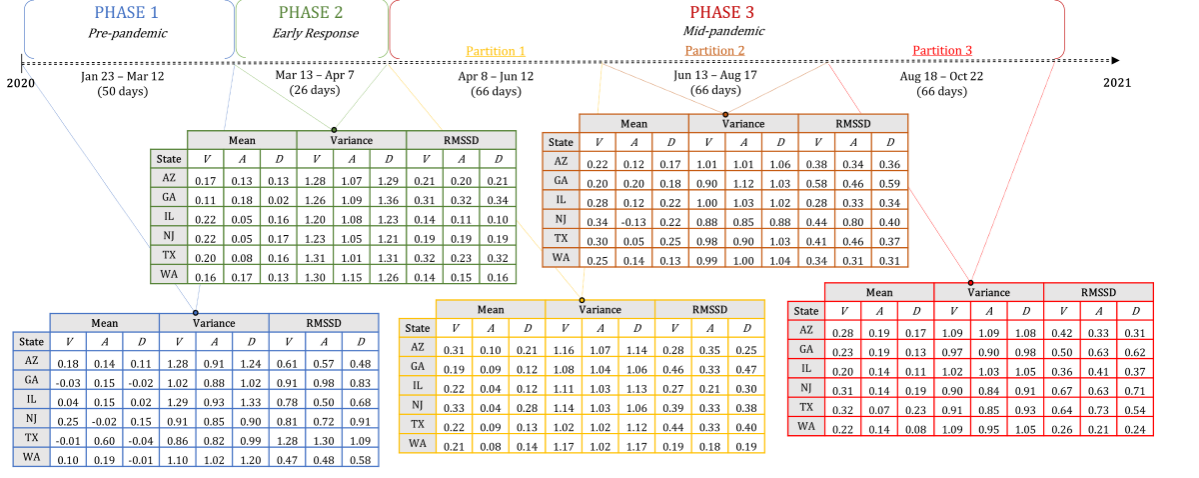
Affect descriptive statistics of representative states across phases of the COVID-19 pandemic. Mean, variance, and RMSSD of daily averaged standardized valence, arousal, and dominance were calculated across news headlines in 6 geographically disparate states. News data were split into 3 distinct phases (with 6 total windows for analysis). A: arousal; D: dominance; RMSSD: root mean square of successive differences; V: valence.

To further illustrate these trajectories of RMSSD in news affect across the country, Figure 4 presents a 3 × 5 grid of percentile-transformed RMSSD choropleths for valence, arousal, and dominance across the 5 predefined time windows of the study. A comparison of affect mean, affect variance, and affect RMSSD in Figures 3 and 5 further emphasizes descriptive statistical similarities and differences across time and state for the 6 representative states. For additional reference, Multimedia Appendix 4 provides all raw statistical results from which time-based descriptions of affect were derived.

### Temporally Agnostic Exploratory Visualization With the Affect Circumplex

**Figure 6:**
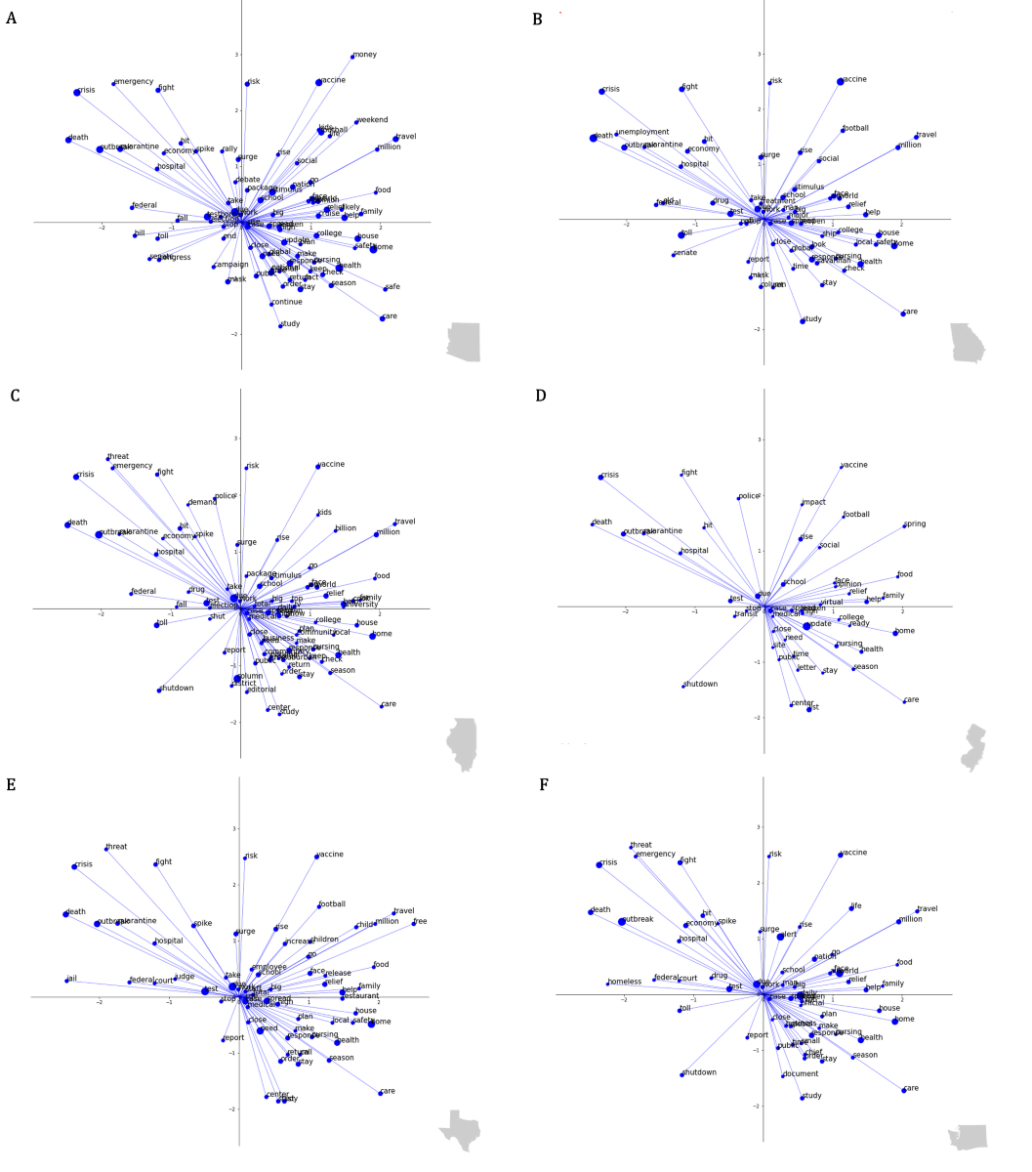
Valence–arousal circumplex of most commonly used words in COVID-19 news headlines across representative states. Valence–arousal circumplex plots of the most frequently used words in COVID-19–related news headlines. Plots are anchored in 2D Euclidean space where the x-axis indicates standardized valence of a word and the y-axis indicates standardized arousal of a word. All represented words reflect a state-specific frequency of use at or above 2SDs from the mean use across all words in the news headlines for the respective state. The size of the data point scales with relative frequency. Words used more frequently are represented by larger points relative to those used less frequently. (A) Arizona, (B) Georgia, (C) Illinois, (D) New Jersey, (E) Texas, and (F) Washington.

With a focus on the VA-2D operationalization of affect dynamics, the resulting circumplex word clouds in Figure 6 highlight interesting time-agnostic similarities and differences in COVID-19–related news headlines across the country. Most generally, descriptive analysis of the 6 representative states indicated an overrepresentation of neutral and positively valenced diction (eg, “health”) with some state-specific variety. In addition, more negatively valenced and strongly arousing language (eg, “emergency”) was less prevalent, however more consistent and ubiquitous across states. The results, in conjunction with those in the “Descriptive Statistics of State-Specific Affect Through Time” section, encourage the implementation of affect-based models that account for state-specific random effects within a temporal framework.

### Generalized Mixed Effects Modeling

Eight different negative binomial mixed effects models (in VA-2D and VAD-3D pairs) were constructed to interrogate the associations between affect dynamics in COVID-19–related news and online mental health–related search term activity. Activity was operationalized in terms of 4 clusters. First, the VA-2D (pseudo-*r*^2^*=*0.11) and VAD-3D (pseudo-*r*^2^*=*0.11) models corresponding to the “All Cluster” of mental health search terms corroborated a statistical significance in the fixed effects of both Spin_VA_ (*P*=.004 and .009, respectively) and Flux_A_ (*P*<.001 and *P*<.001, respectively) with similar respective magnitudes of association (Table 1). Specifically, Spin_VA_ was found to have a significantly (*P*=.004) negative association with mental health search term volume: a unit increase in Spin_VA_ was found to have an approximate 14.8% decrease in mental health search term volume. By contrast, Flux_A_ was found to have a significantly (*P*<.001) positive association with mental health search term volume: a unit increase in Flux_A_ reflects an approximate 24.7% increase in mental health search term counts. For completeness, Multimedia Appendix 6 reports on the conditional modes of the state-based random effects for these models.

**Table 1:** Generalized mixed effects model results for all cluster of mental health search terms^a^.

Fixed effects feature	VA^b^ model				VAD^c^ model		
	β	95% CI	*P* value	β		95% CI	*P* value
Time (*t*)	–.013	–0.035 to 0.009	.26	–.014		–0.036 to 0.009	.23
Valence (*V*)	–.061	–0.126 to 0.004	.07	–.047		–0.136 to 0.043	.31
Arousal (*A*)	–.01	–0.077 to 0.057	.77	–.015		–0.082 to 0.053	.67
Dominance (*D*)				–.034		–0.119 to 0.052	.44
Spin_VA_	–.16	–0.270 to –0.050	.004^d^	–.17		–0.297 to –0.042	.009^d^
Spin_VD_				.01		–0.103 to 0.123	.87
Spin_AD_				.03		–0.092 to 0.151	.63
Pulse_VA_	.131	–0.031 to 0.292	.11	.095		–0.108 to 0.299	.36
Pulse_VD_				–.314		–0.717 to 0.089	.13
Pulse_AD_				.035		–0.179 to 0.249	.75
Flux_V_	.016	–0.084 to 0.115	.76	.318		–0.006 to 0.642	.05
Flux_A_	.221	0.116 to 0.326	<.001^e^	.216		0.106 to 0.325	<.001^e^
Flux_D_				.09		–0.231 to 0.411	.58

^a^Results of the negative binomial mixed effects model for the “All Cluster” summed mental health terms outcome. The VA model includes affect and circumplex features representing the valence and arousal dimensions of words, while the VAD model includes affect and circumplex features representing the valence, arousal, and dominance dimensions of words.

^b^VA: valence–arousal.

^c^VAD: valence–arousal–dominance.

^d^*P*<.05.

^e^*P*<.001.

Second, the VA-2D (pseudo-*r*^2^*=*0.11) and VAD-3D (pseudo-*r*^2^*=*0.11) models corresponding to the “Anxiety Cluster” of mental health search terms suggest a consistent statistically significant (*P*<.001 and *P*<.001, respectively) positive association between Flux_A_ and anxiety-related search term volume, with Flux_V_ (*P=*.04) and *D* (*P*=.03) exhibiting additional significance in the VAD-3D model only (Table 2). Significance (*P*<.001) in Flux_A_ partially echoes findings in the “All Cluster” with a 1 unit increase in Flux_A_ accounting for an approximate 24.5% increase in anxiety-related online search activity. Idiosyncratic to the VAD-3D model, a 1 unit increase in Flux_V_ was associated with an approximately 42.0% increase, while a 1 unit increase in *D* was associated with an approximately 9.1% decrease in anxiety-related search term counts. For completeness, Multimedia Appendix 6 reports on the conditional modes of the state-based random effects for these models.

**Table 2:** Generalized mixed effects model results for anxiety cluster of mental health search terms^a^.

Fixed effects feature	VA^b^ model					VAD^c^ model					
	β	95% CI	*P* value	β			95% CI		*P* value		
Time (*t*)	–0.02	–0.042 to 0.003	.08		–0.023			–0.045 to 0.000		.05	
Valence (*V*)	–0.06	–0.126 to 0.005	.07	–0.003			–0.094 to 0.087		.94		
Arousal (*A*)	–0.016	–0.084 to 0.052	.65	–0.025			–0.094 to 0.044		.48		
Dominance (*D*)				–0.095			–0.181 to –0.008		.03^d^		
Spin_VA_	–0.109	–0.220 to 0.002	.06	–0.11			–0.239 to 0.189		.09		
Spin_VD_				0.007			–0.109 to 0.123		.90		
Spin_AD_				0.036			–0.088 to 0.160		.57		
Pulse_VA_	0.078	–0.085 to 0.241	.35	0.032			–0.173 to 0.238		.76		
Pulse_VD_				–0.357			–0.768 to 0.053		.09		
Pulse_AD_				0.038			–0.178 to 0.254		.73		
Flux_V_	0.006	–0.094 to 0.106	.91	0.351			0.021 to 0.680		.04^d^		
Flux_A_	0.219	0.112 to 0.326	<.001^e^	0.215			0.104 to 0.327		<.001^e^		
Flux_D_				0.092			–0.235 to 0.419		.58		

^a^Results of the negative binomial mixed effects model for the “Anxiety Cluster” summed mental health terms outcome. The VA model includes affect and circumplex features representing the valence and arousal dimensions of words, while the VAD model includes affect and circumplex features representing the valence, arousal, and dominance dimensions of words.

^b^VA: valence–arousal.

^c^VAD: valence–arousal–dominance.

^d^*P*<.05.

^e^*P*<.001.

Third, the VA-2D (pseudo-*r*^2^*=*0.10) and VAD-3D (pseudo-*r*^2^*=*0.10) models corresponding to the “Depression Cluster” of mental health search terms uncovered consistent statistical significance for the fixed effects of Spin_VA_ (*P*<.001 and *P*<.001, respectively) and Flux_A_ (*P*<.001 and *P*<.001, respectively) with additional statistical significance for *V* (*P*=.04) and Pulse_VA_ (*P*=.03) within the VA-2D model only (Table 3). Akin to what was observed in the “All Cluster”, Spin_VA_ was found to have a significantly (*P*=.003) negative association with depression-related search term counts: a unit increase in Spin_VA_ was associated with an 18.7% decrease in depression-related search volume. Likewise, Flux_A_ had a significantly (*P*<.001) positive association with depression-related search activity: there was an approximate 28.9% increase in depression-related search counts for every 1 unit increase in Flux_A_. Idiosyncratic to the VA-2D model, a 1 unit increase in *V* was associated with an approximate 6.9% decrease, while a 1 unit increase in Pulse_VA_ was associated with an approximate 20.2% increase in depression-related search term counts. For completeness, Multimedia Appendix 6 reports on the conditional modes of the state-based random effects for these models.

**Table 3:** Generalized mixed effects model results for depression cluster of mental health search terms^a^.

Fixed effects feature	VA^b^ model				VAD^c^ model			
	β	95% CI	*P* value	β		95% CI	*P* value	
Time (*t*)	–0.015	–0.037 to 0.007	.18	–0.016		–0.039 to 0.007	.17	
Valence (*V*)	–0.071	–0.138 to –0.071	.04^d^	–0.072		–0.164 to 0.020	.13	
Arousal (*A*)	–0.009	–0.077 to 0.059	.80	–0.01		–0.079 to 0.059	.78	
Dominance (*D*)				–0.012		–0.099 to 0.076	.80	
Spin_VA_	–0.207	–0.320 to –0.094	<.001^e^	–0.228		–0.358 to –0.098	<.001^e^	
Spin_VD_				0.015		–0.101 to 0.130	.80	
Spin_AD_				0.048		–0.078 to 0.173	.45	
Pulse_VA_	0.184	0.017 to 0.351	.03^d^	0.138		–0.072 to 0.348	.20	
Pulse_VD_				–0.247		–0.657 to 0.164	.24	
Pulse_AD_				0.052		–0.170 to 0.273	.65	
Flux_V_	0.049	–0.053 to 0.152	.35	0.301		–0.031 to 0.633	.08	
Flux_A_	0.254	0.146 to 0.362	<.001^e^	0.246		0.133 to 0.358	<.001^e^	
Flux_D_				0.041		–0.287 to 0.368	.81	

^a^Results of the negative binomial mixed effects model for the “Depression Cluster” summed mental health terms outcome. The VA model includes affect and circumplex features representing the valence and arousal dimensions of words, while the VAD model includes affect and circumplex features representing the valence, arousal, and dominance dimensions of words.

^b^VA: valence–arousal.

^c^VAD: valence–arousal–dominance.

^d^*P*<.05.

^e^*P*<.001.

At the request of an anonymous reviewer, identical negative binomial mixed effects models with time modeled as logistic were run to challenge the above models’ assumption of time as linearly associated with the outcome. Using Efron pseudo-*r*^2^ to assess comparative fit of the logistic-transformed time models with their respective linear version, results were comparable to 2 decimal places (pseudo-*r*^2^*=*0.10-0.11). As such, treating time as linear or logistic did not lead to an appreciable difference in fit.

**Figure 7:**
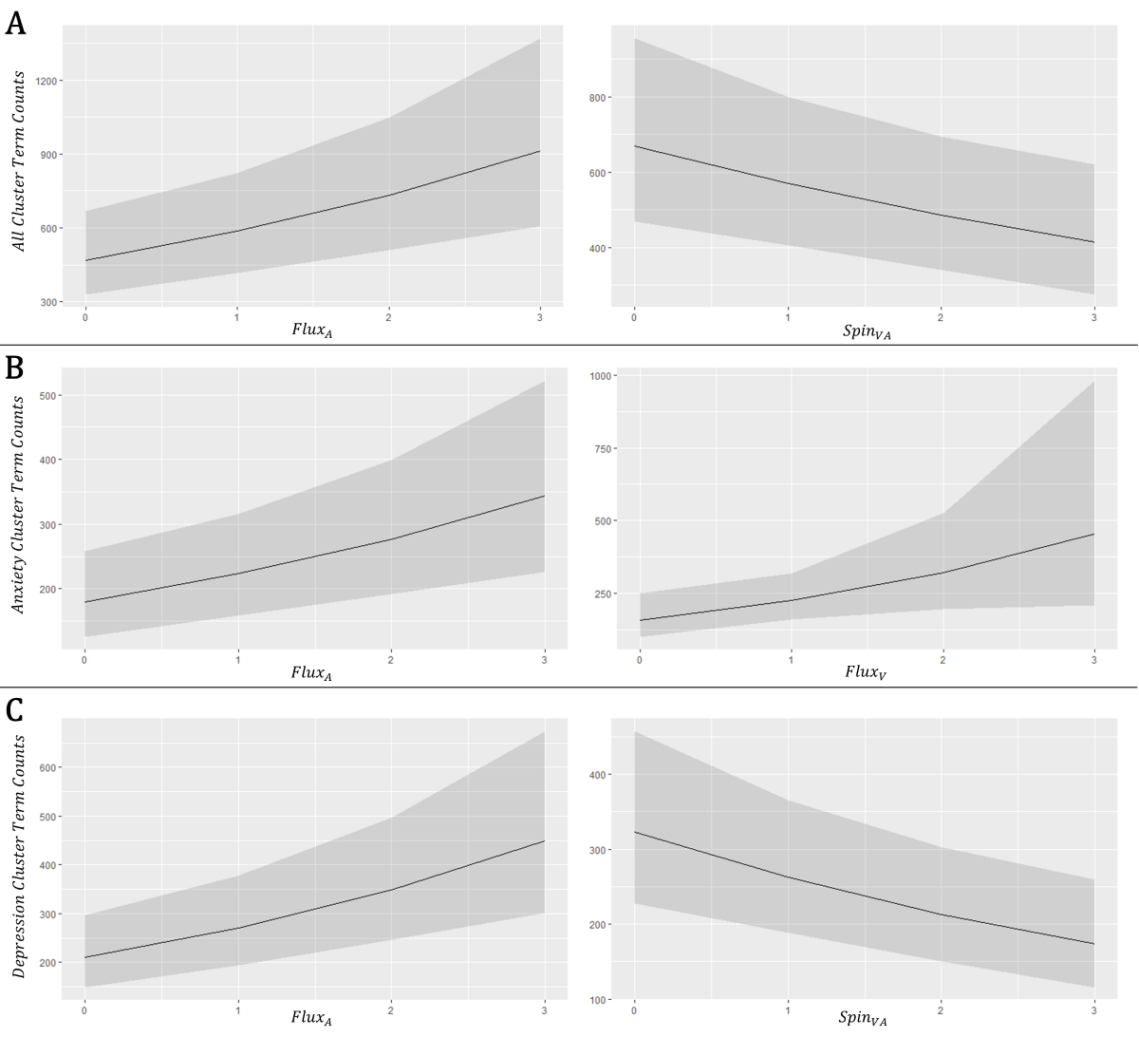
Marginal effect plots of significant VA-2D affect circumplex features on mental health search term counts. The prediction plots illustrate the marginal impact of the most significant affect features on online mental health search term count outcome from the cluster’s respective VA-2D negative binomial mixed model. The x-axis indicates the potential value (0-3) of the designated affect feature, and the y-axis is the model-predicted search term count. Slopes recapitulate the directionalities of association as enumerated in Tables 1-3. (A) “All Cluster”; (B) “Anxiety Cluster”; (C) “Depression Cluster”. VA: valence–arousal. VA-2D: valence-arousal-two-dimensional.

**Figure 8:**
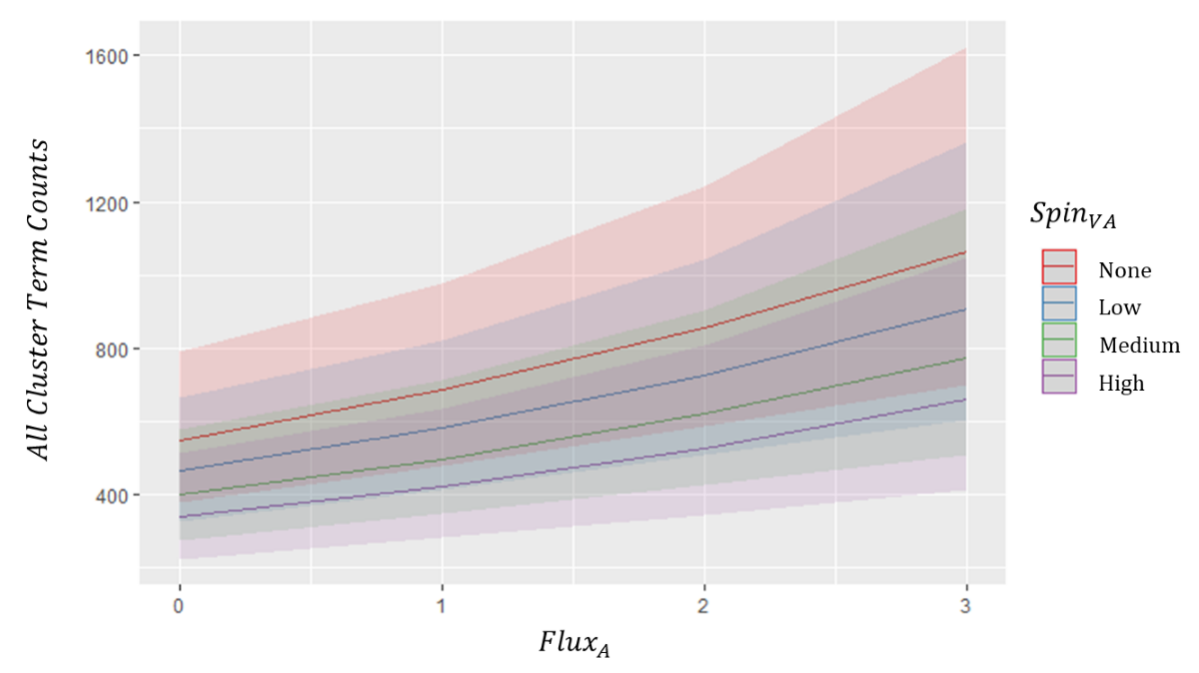
Interaction plot of overall significant affect circumplex features from VA-2D All Cluster model. Illustration of the marginal interactions of the most significant circumplex features from the fit VA-2D circumplex “All Cluster” negative binomial mixed model. Higher Flux_A_ influences predictions toward higher overall activity in online mental health search term counts, while simultaneous higher Spin_VA_ dampens, but does not eliminate, the overall trajectory toward higher search term activity. VA: valence–arousal. VA-2D: valence-arousal-two-dimensional.

Both spin and flux were consistently found to be significant (*P*<.05) in the above mixed models. Given this prominence, Figure 7 first provides an intuitive visualization of the marginal effect of the cluster-/model-specific significant (*P*<.05) spin and flux-based parameters on the frequency of mental health search term clusters, indicating that lower spin and higher flux individually result in larger predicted counts of cluster-related search terms. Considering the significance of Spin_VA_ and Flux_A_ simultaneously, Figure 8 then illustrates their interactive effect on the predicted summative count of all mental health search terms (All Cluster), further implicating overall mental health search term behavior as positively associated with Flux_A_/Flux_V_ and negatively associated with Spin_VA_.

## Discussion

This research endeavor revealed patterns in day-to-day COVID-19 news headline affect variation across the first 9 months of the pandemic. Applying a rule-based, empirical approach to sentiment analysis, time-agnostic similarities and differences in word use and affect were found across the United States, including the ubiquitous use of negatively valenced and strongly arousing language. Initial temporal analyses revealed trends in affect change within and between phases of the pandemic, highlighting the potential importance of operationalizing patterns of expression in future investigations involving written sentiment. For example, the generalized mixed-effects models employed in this work utilized circumplex-based predictors, revealing that an increased consistency in affective tone (Spin_VA_) was predictive of increased depression-related search term activity, and emotional language patterns indicative of affective uncontrollability (Flux_A_) contributed more generally to an increase in online mental health search term frequency.

This study leveraged both news and internet search data to interrogate the association between the daily affective dimensions of local news headlines and online mental health search behavior across the United States. Using the recent COVID-19 pandemic as an example, this research applied the circumplex theory of affect within the context of NLP to operationalize the dynamics of language-based emotion. These operationalizations were then employed within a time-anchored, generalized mixed modeling framework to gauge the significance of the circumplex properties of news headlines on same-day mental health–related search term volume. The study aimed to first explore news coverage temporally, holistically, and geographically from the unique quantitative perspective of word affect (ie, valence, arousal, and dominance). Through basic descriptive statistics and summative data visualization, these efforts profiled key similarities and differences which informed and justified modeling choices for subsequent hypothesis testing. After establishing this descriptive baseline of COVID-19–related news affect, the research was interested in testing hypotheses concerning the relative significance of affect flux, pulse, and spin to ultimately contribute a unique and more nuanced explanation of the semantic mechanisms that underlie the impact of written news exposure on the mental health of society.

As an initial exploratory analysis, this study took daily averaged values of word use affect in COVID-19 news headlines and calculated the state- and pandemic phase–specific mean, variance, and RMSSD of these values. One primary goal of this endeavor was to compare affect coverage through time from both traditional, pattern-agnostic quantifications (ie, mean and variance) with one that began to interrogate notions of affective dynamics (RMSSD within a phase). The results indicated that while the mean and variance of phase-specific COVID-19 news affect did not show significant change through time (Figure 5), there was a clear recapitulation of RMSSD patterns of change across the vast majority of states (Figure 4). Example comparisons of mean and RMSSD in 6 representative states further reflected the nature of these statistical patterns (Figure 3). Taken together, these findings indicated several important aspects of COVID-19 news headline sentiment.

First, and most broadly, these findings indicate that the phenomenology of written sentiment in the context of news coverage is volatile and dynamic; RMSSD can be thought of as an inspiration and justification for applying circumplex-based modeling in subsequent hypothesis testing. Second, deviance among the 50 states indicates that while there is a potentially significant pattern of affect change through time, it does not manifest consistently and to the same degree across the United States. Thus, modeling should account for “state” as a random effect when interrogating any affect–outcome relationship from the data, insinuating the utility of a mixed modeling strategy. Third, these results provide a baseline, descriptive statistical narrative of COVID-19 news coverage throughout the first 9 months of the pandemic. The employment of emotional written language in news, while showing little variance or change in central tendency between pandemic phases holistically (Figures 3A and 5), was characterized by appreciable patterns of change *within each phase* which differ from one phase to the next (Figures 3B(1-6), 4, and 5). Thus, day-to-day fluctuations in news sentiment, as well as the patterns of these fluctuations in local news, may be potentially informative and impactful aspects of news coverage on the resulting mental health of the American people. In general, the initial findings supported the notion that written news affect in this setting is nuanced and thus requires operationalizations and associated modeling that can more subtly capture changes in the dimensions of sentiment through time.

Before any digital surrogates of mental health were incorporated for hypothesis testing, this study sought to additionally provide some linguistic characterization to the headline content driving the aforementioned statistical patterns of sentiment. The resulting word clouds depicting the most commonly used words in COVID-19 news headlines across the 6 representative states aimed not only to interrogate the nuances in state-specific diction, but also to consider the valence and arousal of these headlines at the word level (Figure 6). Words with high arousal and low valence (eg, “emergency,” “quarantine,” and “fight”) were ubiquitous across the majority of the representative states, likely reflecting common COVID-19–related headlines that may not be idiosyncratic to state-specific news outlets and therefore representative of common themes in news coverage across the United States. However, the word clouds of the representative states predominantly consisted of words with high valence and varying arousal. While some of these words suggest a direct connection to COVID-19–related updates (eg, “vaccine,” “travel,” and “stimulus”), others are more indicative of the impact of COVID-19 on everyday life specific to that state (eg, “football,” “college,” and “restaurant”). The over-representation of words with high valence is worthy of note as it may lend to a misinterpretation of COVID-19 news headlines as being generally positive, as reflected by a positive mean valence across the top most commonly used words in a given state. This interpretation highlights the necessity for contextualization of the headline’s diction. For example, when mapped against the valence–arousal circumplex, the headline “Massachusetts nurses, scared for their safety, brace for coronavirus peak” produces 2 words of positive valence (“safety” and “peak”), matched by 2 words of negative valence (“scared” and “brace”; Figure 1A). Despite an inconclusive depiction of the headline’s sentiment when operationalized by the mean valence of the 4 individually mapped words, the headline as a whole suggests a clear sense of fear. Such examples highlight that while the incorporation of mean valence may allow for comparison across a single affect dimension, additional introspection is required to better understand temporal and multidimensional variation in news headline sentiment.

Following the exploratory and descriptive endeavors of COVID-19 news headline coverage, the study turned to the core issue at hand, namely, the interrogation of the impact of these daily news headlines on the mental health–related online search behavior of the US population. Using same-day Google Trends count data of key mental health terms, negative binomial mixed effects models were constructed using time and affect circumplex-derived variables. Valence, arousal, and dominance (ie, flux, pulse, and spin) were used as fixed effects in both VA-2D and VAD-3D representations of word affect along with “state” as a random effect (see the “Generalized Mixed Effects Modeling” section). The results of these circumplex-based modeling efforts can be partially understood through the lens of established psychological theory and are concomitant with word affect patterns that reflect the zeitgeist of the early pandemic. In addition, the novel application of sentiment analysis within a circumplex framework has highlighted several novel foci for future research consideration.

One key methodological feature of the hypothesis-driven mixed modeling was the summation of daily state-specific mental health search term count activity by 4 clusters: (1) All, (2) Depression, (3) Anxiety, and (4) Nonspecific (see the “Mental Health Search Activity” and “Generalized Mixed Effects Modeling” sections). For terms related to the construct of depression, the models implicate both spin in the valence–arousal circumplex (Spin_VA_) and flux–arousal (Flux_A_) as statistically significant predictors of depression-related search term activity through time in both the VA-2D and VAD-3D representations of affect (Table 3). This result indicates that both the affective position and magnitude of words in news headline exposure were associated with depression-related search activity. Moreover, this result differed from the results of the “Anxiety” cluster models, where only Flux_A_ was a statistically significant predictor of anxiety-related search activity in both the VA-2D and VAD-3D versions of the model. The exclusive significance of Flux_A_ in the anxiety models implies that only the magnitude of the arousal dimension is important for prediction when anxiety-related terms are considered (Table 2). Additionally, the “All Cluster” models, where anxiety, depression, and nonspecific search terms were considered, reflected the union of circumplex variable significance for the “Anxiety”, “Depression”, and “Nonspecific” (not-shown; no significance) clusters (Table 1). When considering the importance of spin for depression-related search behavior, Spin_VA_ can be thought of as representing affective “tone” or “mood” (ie, the “kind” of affect [37]) due to its solely position-based (and not magnitude-based) operationalization within the circumplex. Thus, the finding that lower spin (more consistent tone) is associated with higher depression-related search term activity may be a reflection of the mundane, of a situation that is stagnant and inescapable, thereby potentially fueling depressive thoughts and feelings. Such a description echoes feelings of languishing—a void between depression and peak psychological well-being—that have widely characterized the mental milieu of those affected by the COVID-19 pandemic [51].

Turning now to the significance of Flux_A_ in the “Anxiety Cluster,” “Depression Cluster,” and “All Cluster” models, the learned helplessness hypothesis may offer some explanation. The learned helplessness hypothesis describes a situation where perceived uncontrollable events lead to a learned disconnection between behavior and outcome, ultimately producing motivational, cognitive, and emotional states of uncontrollability during this learning process [52]. This framing purports that uncontrollable aversive events produce greater emotional disruption than controllable aversive events, which ultimately leads to what could be described as a “defeatist” mentality (of particular note for depression). The significance of Flux_A_ as a predictor of mental health search term counts highlights the affective uncontrollability of COVID-19 reporting (and COVID-19 itself); ubiquitous feelings of uncertainty surrounding the pandemic have been parroted by the news, thereby reinforcing these sentiments through time. The positive directionality of association between Flux_A_ and the “Anxiety Cluster,” “Depression Cluster,” and “All Cluster” model outcomes is indicative of the impact of greater-expressed uncertainty on the mental health of the population.

In joint consideration of low spin and high flux, with importance centered on valence and arousal, the modeling results showed that COVID-19–related news coverage through time clashes low energy and high energy emotional states, a combination (and fluctuation) of both the arousing and the mundane. Given the behavioral and environmental context and uncertainties surrounding the pandemic, this dynamic may be particularly disturbing for consumers and may engender heightened feelings of both depression and anxiety, ultimately exacerbating mental health issues. Figures 7 and 8 offer some preliminary empirical support for this association.

The application and similarity of results between the VA-2D and VAD-3D models of affect support the overall robusticity of the results. Such a general recapitulation also implies that dominance, in the setting of news headlines, does not seem to contribute to modeling the association between written news affect and mental health search term behavior; the VA-2D circumplex statistically accounts for mental health search term behavior on its own. However, there are some discrepancies worth noting. In modeling anxiety-related search terms, flux valence (Flux_V_) is significant in the VAD-3D model only, meaning the dynamics of valence become important only after controlling for dominance. This supports a common critique of the 2D circumplex, which states that the limited framing may not adequately capture nuances in emotion. For example, nervousness and anger share a similar space in the VA circumplex (negative valence, positive arousal), but with the addition of dominance in the VAD circumplex, nervousness (negative dominance) and anger (positive dominance) become separable in 3D space. While the current results saw limited instances of discrepancy between the 2, it is worth noting for future endeavors that accounting for dominance may uncover nuanced differences in the affective properties and associated circumplex dynamics that exist among words and their corresponding emotional states. Dimensionality notwithstanding, this study has underlined arousal (over both valence and dominance) as the most significant affective dimension in consideration of mental health search behavior outcomes. The implications of the significance of arousal are not clear and should be a focus for future work in this area.

This study contributes to a small yet growing body of literature connecting NLP, sentiment analysis, and affective dynamics, ultimately offering a novel framework to more finely interrogate the impact of emotion in written form. Leveraging a large and robust data set of time-anchored news headlines and internet search behavior, theory-driven operationalizations of emotion, along with empirically informed modeling of association against the backdrop of a widespread emotional and impactful stimulus, this work serves both as a theoretical proof of concept for methodological integration and as a practical, big data–driven investigation into the impact of language on the mental health of society. While acknowledging these strengths, this study also has several limitations worth noting when considering the significance of the results. First and foremost, the analyses only extracted individual words, or unigrams, from news headlines and used a reference dictionary to quantify the sentiment of each word. While considering unigrams is convenient for extracting the sentiment of individual words, they do not take into account contextual elements of full sentences that may disambiguate the meaning and therefore sentiment of a word [53]. For instance, the word “good” can be modified with an intensifier like “very” to increase its positive sentiment or a negator like “not” to give it a negative sentiment [54]. Thus, using an *n*-gram approach, where *n* is a number of words greater than 1 (eg, bigrams, trigrams), may have yielded more accurate quantifications of sentiment contained within the COVID-19–related news headlines. However, because this study utilized an externally developed affect reference dictionary for English lemmas, which mapped unigrams to sentiments, the authors of this study would have had to empirically derive a new reference dictionary for *n*-grams of a comparable quality to the one used. In addition, because the external reference dictionary could only account for 34.47% (252,254/731,607) of the lemmas extracted from the COVID-19–related news headlines, the majority of the extracted lemmas could not be considered for analysis. Second, this study relied on the Media Cloud API client for COVID-19–related headline availability. It is possible that some news stories were not available in the database and therefore not included in this analyses. While the news headline corpus was extensive in totality, state-wise news coverage was dependent on database availability and thus not equally representative nor exhaustive. Finally, the present methods used approximate calculations of mental health–related term frequencies based on normalized Google Trends values, as Google Trends does not provide absolute search term counts. These estimated frequencies were conservative, consistent, and empirically based, and thus the impact of using them on the present results was likely slim; however, the true search term counts may yield slightly different results.

This work demonstrated the general promise of leveraging the circumplex model of affect to written content, providing an example for how circumplex theory can be integrated with NLP and sentiment analysis techniques. The results of this work operationalized sentiment analysis via the theory-driven affect circumplex to uncover nuanced dynamics of word use, thus suggesting that such a combination of analytical tools is uniquely informative and promising for analyzing associations relating to emotional dynamics, especially within a longitudinal context. Future efforts may benefit from applying an expanded sentiment reference dictionary or machine learning–based sentiment analysis approaches with the inclusion of *n*-grams to more fully test the practical application and theoretical capabilities of the circumplex model of affect on text-based data. Furthermore, in analyzing news headline content, the present results implicated arousal as the most informative and statistically significant circumplex dimension. Future studies of news content not necessarily limited to COVID-19 or mental health may therefore find it informative to incorporate and focus attention on the arousal-based qualities of word use.

## Appendix

Multimedia Appendix 1 Table of news outlets and associated details.

Multimedia Appendix 2 Table of Google Trends comparator search terms by date.

Multimedia Appendix 3 Table of additional stopwords used in news story title preprocessing.

Multimedia Appendix 4 The raw statistical results of state-specific affect mean, affect variance, and affect RMSSD across the five temporal windows (three pandemic phases) of the study. Affect is quantified in three dimensions: (i) valence, (ii) arousal, and (iii) dominance.

Multimedia Appendix 5 The tabular, long-format data used in negative binomial mixed-modeling to model the association between the affective dynamics of state-specific COVID news headlines (via the affect circumplex) on same-day online mental health search term activity.

Multimedia Appendix 6 Choropleths of state-based random effects from negative binomial mixed models. The conditional modes for each state are plotted for each of the six models. Darker blue coloration denotes increasingly positive associations with the outcome, while darker red coloration denotes increasingly negative associations with the outcome.

## Abbreviations

API: application programming interface
NLP: natural language processing
RMSSD: root mean square of successive differences
VA: valence–arousal
VAD: valence–arousal–dominance
