# Supplementary material for: Using the COVID-19 Pandemic to Assess the Influence of News Affect on Online Mental Health-Related Search Behavior Across the United States: Integrated Sentiment Analysis and the Circumplex Model of Affect
Source: J Med Internet Res. 2022 Jan 27;24(1):e32731. doi: 10.2196/32731 (PMC8805454; doi:10.2196/32731)
Supplement: Multimedia Appendix 1 [file jmir_v24i1e32731_app1.doc]

Supplementary Table 1

*News Outlets and Associated Details*

| **State** | **News Outlet Name** | **Publication City** | **Media Cloud ID** | **Total News**  **Stories** | **Web Traffic Rank*** | **State Circulation Rank*** |
| --- | --- | --- | --- | --- | --- | --- |
| Alabama (AL) | Montgomery Advertiser | Montgomery | 28907 | 324 | 1 | 9 |
|  | Tuscaloosa News | Tuscaloosa | 29130 | 197 | 2 | 6 |
|  | Dothan Eagle | Dothan | 160783 | 12 | 3 | 8 |
| Alaska (AK) | Anchorage Daily News | Anchorage | 25064 | 311 | 1 | NR |
|  | Juneau Empire | Juneau | 74063 | 1 | 2 | 5 |
|  | Daily News-Miner | Fairbanks | 29366 | 2 | 3 | 3 |
| Arizona (AZ) | The Arizona Republic | Phoenix | 649899 | 3108 | 1 | 1 |
|  | Phoenix New Times | Phoenix | 27200 | 111 | 3 | NR |
|  | The Arizona Daily Sun | Flagstaff | 26100 | 70 | 4 | 6 |
| Arkansas (AR) | Arkansas Times | Little Rock | 77657 | 240 | 2 | 4 |
|  | Southwest Times Record | Fort Smith | 81134 | 88 | 4 | 1 |
| California (CA) | Los Angeles Times | Los Angeles | 27359 | 1280 | 1 | 1 |
|  | The Examiner | San Francisco | 28006 | 178 | 3 | 5 |
|  | San Jose Mercury News | San Jose | 27854 | 1438 | 4 | 2 |
| Colorado (CO) | The Denver Post | Denver | 31 | 832 | 1 | 1 |
|  | The Gazette | Colorado Springs | 78756 | 153 | 2 | 3 |
|  | Westword | Denver | 83103 | 143 | 3 | NR |
| Connecticut (CT) | Hartford Courant | Hartford | 59 | 369 | 1 | 2 |
|  | New Haven Register | New Haven | 32270 | 674 | 3 | 6 |
|  | The Stamford Advocate | Stamford | 25411 | 624 | 4 | 7 |
| Delaware (DE) | The News Journal | New Castle | 20465 | 643 | 1 | NR |
|  | Delaware State News | Dover | 312220 | 228 | 2 | 4 |
| Florida (FL) | Palm Beach Post | Palm Beach | 60 | 2122 | 5 | 5 |
|  | South Florida Sun-Sentinel | Fort Lauderdale | 649920 | 1078 | 2 | 3 |
|  | Orlando Sentinel | Orlando | 38 | 1147 | 3 | 2 |
| Georgia (GA) | Savannah Morning News | Savannah | 79999 | 1098 | 2 | 4 |
|  | Athens Banner-Herald | Athens | 35424 | 1128 | 4 | 10 |
|  | Atlanta Journal-Constitution | Atlanta | 17 | 405 | 1 | 1 |
| Hawaii (HI) | Star Advertiser | Honolulu | 39573 | 2106 | 1 | 2 |
|  | West Hawaii Today | Kailua Kona | 225040 | 327 | 2 | NR |
| Idaho (ID) | Idaho Press-Tribune | Nampa | 69266 | 33 | 4 | 6 |
|  | The Times-News | Twins Falls | 113316 | 76 | 2 | 2 |
| Illinois (IL) | Chicago Tribune | Chicago | 9 | 3025 | 1 | 1 |
|  | Daily Herald | Arlington Heights | 104 | 1642 | 2 | 3 |
|  | Chicago Reader | Chicago | 32385 | 22 | 3 | NR |
| Indiana (IN) | The Indianapolis Star | Indianapolis | 85390 | 682 | 1 | 1 |
|  | The Times | Munster | 71797 | 84 | 2 | 3 |
|  | South Bend Tribune | South Bend | 27205 | 41 | 3 | 2 |
| Iowa (IA) | The Des Moines Register | Des Moines | 18170 | 580 | 1 | 1 |
|  | The Gazette | Cedar Rapids | 18171 | 477 | 3 | 2 |
| Kansas (KS) | The Topeka Capital Journal | Topeka | 19017 | 1187 | 2 | 4 |
| Kentucky (KY) | The Courier-Journal | Louisville | 91426 | 1082 | 1 | 1 |
|  | The Daily News | Bowling Green | 144609 | 26 | 3 | 4 |
| Louisiana (LA) | The Advocate | Baton Rouge | 367906 | 160 | 1 | 1 |
|  | The Times | Shreveport | 81549 | 286 | 2 | 6 |
|  | The News Star | Monroe | 90472 | 212 | 4 | 5 |
| Maine (ME) | Portland Press Herald | Portland | 366984 | 1084 | 1 | 1 |
|  | Bangor Daily News | Bangor | 20320 | 1267 | 2 | 2 |
|  | Kennebec Journal / Morning Sentinel | Augusta | 25755 | 310 | 4 | 7 |
| Maryland (MD) | The Baltimore Sun | Baltimore | 34 | 687 | 1 | 1 |
|  | The Capital Gazette | Annapolis | 70310 | 160 | 2 | 5 |
|  | The Frederick News-Post | Frederick | 81618 | 17 | 3 | NR |
| Massachusetts (MA) | The Boston Globe | Boston | 15 | 1599 | 2 | 2 |
|  | The Boston Herald | Boston | 51 | 2221 | 3 | 4 |
|  | The Republican | Springfield | 24649 | 1146 | 4 | 3 |
| Michigan (MI) | The Detroit Free Press | Detroit | 20262 | 986 | 1 | 2 |
|  | The Detroit News | Detroit | 25798 | 1338 | 2 | 1 |
|  | Lansing State Journal | Lansing | 79287 | 466 | 3 | 7 |
| Minnesota (MN) | Star Tribune | Minneapolis | 19 | 2186 | 1 | 1 |
|  | Pioneer Press | St. Paul | 52 | 927 | 2 | 2 |
|  | Duluth News-Tribune | Duluth | 86929 | 664 | 3 | 3 |
| Mississippi (MS) | The Clarion-Ledger | Jackson | 54342 | 373 | 1 | 1 |
|  | The Daily Journal | Tupelo | 70602 | 20 | 3 | NR |
|  | Hattiesburg American | Hattiesburg | 72156 | 359 | 4 | 9 |
| Missouri (MO) | St. Louis Post-Dispatch | St. Louis | 27 | 2316 | 1 | 1 |
|  | Springfield News-Leader | Springfield | 26563 | 346 | 3 | 3 |
|  | The Columbia Daily Tribune | Columbia | 27134 | 415 | 5 | 5 |
| Montana (MT) | Billings Gazette | Billings | 29602 | 49 | 1 | 1 |
|  | Missoulian | Missoula | 29628 | 17 | 2 | 3 |
|  | Great Falls Tribune | Great Falls | 87252 | 128 | 3 | 2 |
| Nebraska (NE) | Omaha World-Herald | Omaha | 54 | 288 | 1 | 2 |
|  | Lincoln Journal-Star | Lincoln | 21794 | 64 | 2 | 3 |
|  | The Grand Island Independent | Grand Island | 88101 | 27 | 3 | 4 |
| Nevada (NV) | Las Vegas Review-Journal | Las Vegas | 64 | 823 | 1 | 1 |
|  | Reno Gazette-Journal | Reno | 29476 | 440 | 3 | 3 |
|  | Nevada Appeal | Carson City | 25878 | 86 | 4 | 9 |
| New Hampshire (NH) | New Hampshire Union Leader | Manchester | 366646 | 26 | 1 | 1 |
|  | Concord Monitor | Concord | 29410 | 219 | 2 | 4 |
|  | Portsmouth Herald | Portsmouth | 662028 | 781 | 3 | 7 |
| New Jersey (NJ) | The Record | Hackensack | 65 | 1569 | 1 | 7 |
|  | Asbury Park Press | Neptune | 84895 | 958 | 2 | 2 |
|  | Courier-Post | Cherry Hill | 21294 | 563 | 4 | 4 |
| New Mexico (NM) | Albuquerque Journal | Albuquerque | 99 | 765 | 1 | 1 |
|  | Santa Fe New Mexican | Santa Fe | 80065 | 61 | 2 | 3 |
|  | Las Cruces Sun-News | Las Cruces | 78708 | 378 | 3 | 4 |
| New York (NY) | Newsday | Melville | 13 | 1673 | 5 | 3 |
| North Carolina (NC) | The Winston-Salem Journal | Winston-Salem | 32968 | 43 | 4 | 4 |
| North Dakota (ND) | The Forum | Fargo | 98987 | 1116 | 1 | 2 |
|  | The Grand Forks Herald | Grand Forks | 112564 | 903 | 2 | 3 |
|  | Bismarck Tribune | Bismarck | 29275 | 40 | 3 | 1 |
| Ohio (OH) | The Columbus Dispatch | Columbus | 41 | 648 | 2 | 2 |
|  | The Cincinnati Enquirer | Cincinnati | 26590 | 1699 | 3 | 3 |
|  | The Blade | Toledo | 83 | 457 | 4 | 10 |
| Oklahoma (OK) | The Oklahoman | Oklahoma City | 43 | 1705 | 2 | 1 |
|  | The Norman Transcript | Norman | 92026 | 20 | 3 | 8 |
|  | The Oklahoma Daily | Norman | 132908 | 36 | 4 | NR |
| Oregon (OR) | The Oregonian | Portland | 76546 | 77 | 1 | 1 |
|  | Statesman Journal | Salem | 30458 | 444 | 2 | 3 |
|  | The Bulletin | Bend | 22935 | 31 | 3 | 2 |
| Pennsylvania (PA) | Pittsburgh Post-Gazette | Pittsburgh | 45 | 412 | 1 | 2 |
|  | The Patriot-News | Harrisburg | 18920 | 1040 | 3 | 4 |
|  | The Tribune-Review | Greensburg | 26214 | 2295 | 4 | 3 |
| Rhode Island (RI) | The Providence Journal | Providence | 68874 | 1529 | 1 | 1 |
|  | The Newport Daily News | Newport | 662950 | 1140 | 4 | 3 |
| South Carolina (SC) | The Post and Courier | Charleston | 19590 | 381 | 2 | 2 |
|  | The Greenville News | Greenville | 100401 | 474 | 3 | 4 |
| South Dakota (SD) | The Argus Leader | Sioux Falls | 29444 | 572 | 1 | 3 |
|  | Rapid City Journal | Rapid City | 35608 | 48 | 2 | 5 |
|  | The Daily Republic | Mitchell | 32194 | 542 | 4 | 6 |
| Tennessee (TN) | News-Sentinel | Knoxville | 89 | 529 | 2 | 1 |
|  | The Commercial Appeal | Memphis | 71 | 683 | 3 | 4 |
|  | Chattanooga Times Free Press | Chattanooga | 25407 | 599 | 4 | 2 |
| Texas (TX) | Houston Chronicle | Houston | 10 | 981 | 1 | 2 |
|  | The Dallas Morning News | Dallas | 12 | 754 | 2 | 1 |
|  | San Antonio Express News | San Antonio | 33 | 460 | 3 | 6 |
| Utah (UT) | The Salt Lake Tribune | Salt Lake City | 78 | 1413 | 1 | 1 |
|  | The Daily Herald | Provo | 21148 | 24 | 2 | 9 |
|  | Deseret News | Salt Lake City | 20270 | 1540 | 4 | 2 |
| Vermont (VT) | The Burlington Free Press | Burlington | 33500 | 306 | 1 | 1 |
|  | The Rutland Herald | Rutland | 26310 | 3 | 2 | 3 |
|  | Brattleboro Reformer | Brattleboro | 80237 | 23 | 3 | NR |
| Virginia (VA) | Richmond-Times Dispatch | Richmond | 192429 | 141 | 2 | 2 |
|  | Daily Press | Newport News | 26793 | 315 | 4 | 4 |
|  | The Roanoke Times | Roanoke | 21909 | 263 | 5 | 3 |
| Washington (WA) | The Seattle Times | Seattle | 24940 | 4350 | 1 | 1 |
|  | Seattle Post-Intelligencer | Seattle | 32770 | 2000 | 2 | NR |
|  | The Spokesman-Review | Spokane | 26874 | 180 | 3 | 3 |
| West Virginia (WV) | The Herald Dispatch | Huntington | 92445 | 33 | 2 | 1 |
|  | The Register- Herald | Beckley | 33282 | 8 | 3 | 3 |
|  | Gazette-Mail | Charleston | 269122 | 46 | 1 | NR |
| Wisconsin (WI) | Green Bay Press-Gazette | Green Bay | 25451 | 831 | 3 | 7 |
|  | The Journal-Times | Racine | 80754 | 65 | 5 | 8 |
| Wyoming (WY) | Casper Star-Tribune | Casper | 80292 | 100 | 1 | 1 |
|  | The News Record | Gillette | 181501 | 8 | 3 | 5 |
|  | The Sheridan Press | Sheridan | 183082 | 36 | 5 | 6 |
