# Supplementary material for: Using the COVID-19 Pandemic to Assess the Influence of News Affect on Online Mental Health-Related Search Behavior Across the United States: Integrated Sentiment Analysis and the Circumplex Model of Affect
Source: J Med Internet Res. 2022 Jan 27;24(1):e32731. doi: 10.2196/32731 (PMC8805454; doi:10.2196/32731)
Supplement: Multimedia Appendix 2 [file jmir_v24i1e32731_app2.doc]

Supplementary Table 2

*Google Trends Comparator Search Terms by Date*

| **Date** | **Term** | **Estimated Search Volume (in thousands)** |
| --- | --- | --- |
| 03/24/2020 | Tsunami | 50 - 100 |
| 03/25/2020 | Neil Gaiman | 50 - 100 |
| 03/26/2020 | Nintendo Direct | 50 - 100 |
| 03/27/2020 | Julia Garner | 100 - 200 |
| 03/28/2020 | Penguin | 50 - 100 |
| 03/29/2020 | Nier Replicant | 50 - 100 |
| 03/30/2020 | Instacart strike | 100 - 200 |
| 03/31/2020 | DaBaby | 50 - 100 |
| 04/01/2020 | David Blaine | 50 - 100 |
| 04/02/2020 | LaMelo Ball | 50 - 100 |
| 04/03/2020 | Tom Holland | 100 - 200 |
| 04/04/2020 | Joe Rogan | 50 - 100 |
| 04/05/2020 | Drew McIntyre | 50 - 100 |
| 04/06/2020 | Kenny Rogers | 100 - 200 |
| 04/07/2020 | Lele Pons | 50 - 100 |
| 04/08/2020 | Tyler Perry | 50 - 100 |
| 04/09/2020 | Holy Thursday | 50 - 100 |
| 04/10/2020 | Jerome Adams | 50 - 100 |
| 04/11/2020 | Sandra Lee | 50 - 100 |
| 04/12/2020 | Mark Cuban | 50 - 100 |
| 04/13/2020 | Chattanooga tornado | 100 - 200 |
| 04/14/2020 | New Amsterdam | 50 - 100 |
| 04/15/2020 | Jackie Robinson | 50 - 100 |
| 04/16/2020 | Utah earthquake | 50 - 100 |
| 04/17/2020 | Bosch | 50 - 100 |
| 04/18/2020 | Kacey Musgraves | 100 - 200 |
| 04/19/2020 | Freddie Mercury | 50 - 100 |
| 04/20/2020 | GasBuddy | 50 - 100 |
| 04/21/2020 | Val Kilmer | 100 - 200 |
| 04/22/2020 | Weather radar | 100 - 200 |
| 04/23/2020 | Elizabeth Warren | 100 - 200 |
| 04/24/2020 | Velveteen Dream | 50 - 100 |
| 04/25/2020 | Alex Highsmith | 50 - 100 |
| 04/26/2020 | The Last Kingdom | 100 - 200 |
| 04/27/2020 | Viola Davis | 100 - 200 |
| 04/28/2020 | Alex Smith | 50 - 100 |
| 04/29/2020 | TSLA stock | 50 - 100 |
| 04/30/2020 | Mason Rudolph | 50 - 100 |
| 05/01/2020 | NASCAR | 50 - 100 |
| 05/02/2020 | Blue Angels | 50 - 100 |
| 05/03/2020 | Adele | 50 - 100 |
| 05/04/2020 | Francie Frane | 50 - 100 |
| 05/05/2020 | Jerry Seinfeld | 50 - 100 |
| 05/06/2020 | Brendan Leipsic | 50 - 100 |
| 05/07/2020 | Raiders schedule 2020 | 100 - 200 |
| 05/08/2020 | GTA 6 | 50 - 100 |
| 05/09/2020 | Jordan 1 | 50 - 100 |
| 05/10/2020 | Zion Williamson | 50 - 100 |
| 05/11/2020 | Amy Schumer | 50 - 100 |
| 05/12/2020 | YouTube channel | 50 - 100 |
| 05/13/2020 | Mac McClung | 50 - 100 |
| 05/14/2020 | YAP | 50 - 100 |
| 05/15/2020 | Polo G | 50 - 100 |
| 05/16/2020 | Terraria | 50 - 100 |
| 05/17/2020 | Bayern Munich | 50 - 100 |
| 05/18/2020 | Ronan Farrow | 50 - 100 |
| 05/19/2020 | Bon Jovi | 100 - 200 |
| 05/20/2020 | Matt Lauer | 50 - 100 |
| 05/21/2020 | Tony Hawk | 50 - 100 |
| 05/22/2020 | Joe Flacco | 50 - 100 |
| 05/23/2020 | Ray Porter | 50 - 100 |
| 05/24/2020 | Indy 500 | 50 - 100 |
| 05/25/2020 | John Cena | 50 - 100 |
| 05/26/2020 | World of Dance | 50 - 100 |
| 05/27/2020 | Tuesday Morning | 50 - 100 |
| 05/28/2020 | Amazon down | 50 - 100 |
| 05/29/2020 | Nina Dobrev | 100 - 200 |
| 05/30/2020 | Oakland | 100 - 200 |
| 05/31/2020 | Keith Ellison | 100 - 200 |
| 06/01/2020 | Virgil Abloh | 200- 500 |
| 06/02/2020 | Fuller House | 100 - 200 |
| 06/03/2020 | Esper | 100 - 200 |
| 06/04/2020 | Fentanyl | 50 - 100 |
| 06/05/2020 | Roger Goodell | 50 - 100 |
| 06/06/2020 | Chase Hooper | 50 - 100 |
| 06/07/2020 | Cristobal storm | 100 - 200 |
| 06/08/2020 | Ben Platt | 100 - 200 |
| 06/09/2020 | Renee Gracie | 50 - 100 |
| 06/10/2020 | Jon Ossoff | 50 - 100 |
| 06/11/2020 | Jefferson Davis | 50 - 100 |
| 06/12/2020 | Twenty One Pilots | 50 - 100 |
| 06/13/2020 | Starlink | 50 - 100 |
| 06/14/2020 | Persona 4 Golden | 50 - 100 |
| 06/15/2020 | Howard Stern | 50 - 100 |
| 06/16/2020 | Ford Bronco | 50 - 100 |
| 06/17/2020 | Pablo Alboran | 50 - 100 |
| 06/18/2020 | Paul McCartney | 50 - 100 |
| 06/19/2020 | Solar Eclipse | 100 - 200 |
| 06/20/2020 | AMBER Alert | 50 - 100 |
| 06/21/2020 | Shane Dawson | 50 - 100 |
| 06/22/2020 | Michael Keaton | 100 - 200 |
| 06/23/2020 | The Rock | 50 - 100 |
| 06/24/2020 | Larry Nassar | 50 - 100 |
| 06/25/2020 | Tanzanite | 50 - 100 |
| 06/26/2020 | Zoox | 50 - 100 |
| 06/27/2020 | Manchester United | 50 - 100 |
| 06/28/2020 | Brendon Todd | 50 - 100 |
| 06/29/2020 | Lyda Krewson | 50 - 100 |
| 06/30/2020 | Juventus | 50 - 100 |
| 07/01/2020 | Flying snakes | 50 - 100 |
| 07/02/2020 | Warrior Nun | 100 - 200 |
| 07/03/2020 | Claudia Conway | 50 - 100 |
| 07/04/2020 | Black Eyed Peas | 50 - 100 |
| 07/05/2020 | JK Rowling | 50 - 100 |
| 07/06/2020 | Dogecoin | 100 - 200 |
| 07/07/2020 | Ringo Starr | 100 - 200 |
| 07/08/2020 | Ivy League | 50 - 100 |
| 07/09/2020 | Gary Larson | 50 - 100 |
| 07/10/2020 | Far Cry 6 | 50 - 100 |
| 07/11/2020 | Liverpool | 50 - 100 |
| 07/12/2020 | Tesla Model Y | 50 - 100 |
| 07/13/2020 | PS5 pre-order | 50 - 100 |
| 07/14/2020 | Clarisonic | 50 - 100 |
| 07/15/2020 | Mariah Carey | 50 - 100 |
| 07/16/2020 | NFLX stock | 50 - 100 |
| 07/17/2020 | Mortgage rates | 50 - 100 |
| 07/18/2020 | Tony Finau | 50 - 100 |
| 07/19/2020 | The Alienist | 50 - 100 |
| 07/20/2020 | Natalie Maines | 50 - 100 |
| 07/21/2020 | Snap stock | 50 - 100 |
| 07/22/2020 | Selena Gomez | 50 - 100 |
| 07/23/2020 | Kia Telluride | 50 - 100 |
| 07/24/2020 | Outer Banks | 100 - 200 |
| 07/25/2020 | Tom Thibodeau | 50 - 100 |
| 07/26/2020 | Hurricane Douglas update | 50 - 100 |
| 07/27/2020 | Gold price | 50 - 100 |
| 07/28/2020 | Last Chance U | 50 - 100 |
| 07/29/2020 | Joey Bosa | 50 - 100 |
| 07/30/2020 | NASA | 100 - 200 |
| 07/31/2020 | Harvey Updyke | 50 - 100 |
| 08/01/2020 | NHL Playoffs | 50 - 100 |
| 08/02/2020 | El Marro | 50 - 100 |
| 08/03/2020 | Earthquake California | 50 - 100 |
| 08/04/2020 | National Grid | 100 - 200 |
| 08/05/2020 | Instagram Reels | 50 - 100 |
| 08/06/2020 | An American Pickle | 50 - 100 |
| 08/07/2020 | Rob Lowe | 50 - 100 |
| 08/08/2020 | Flyers | 50 - 100 |
| 08/09/2020 | Scottie Scheffler | 50 - 100 |
| 08/10/2020 | MidAmerican | 100 - 200 |
| 08/11/2020 | Raymond Allen | 50 - 100 |
| 08/12/2020 | Sarah Cooper | 50 - 100 |
| 08/13/2020 | Spurs | 50 - 100 |
| 08/14/2020 | Legend of Korra | 50 - 100 |
| 08/15/2020 | Linda Manz | 50 - 100 |
| 08/16/2020 | Hudson River | 50 - 100 |
| 08/17/2020 | Microsoft Flight Simulator 2020 | 100 - 200 |
| 08/18/2020 | 19th Amendment | 50 - 100 |
| 08/19/2020 | Death on the Nile | 50 - 100 |
| 08/20/2020 | Alissa Turney | 50 - 100 |
| 08/21/2020 | Miami Heat | 50 - 100 |
| 08/22/2020 | Snyder Cut | 100 - 200 |
| 08/23/2020 | Convalescent plasma | 100 - 200 |
| 08/24/2020 | Dallas Mavericks | 100 - 200 |
| 08/25/2020 | New World | 100 - 200 |
| 08/26/2020 | Jack Brewer | 100 - 200 |
| 08/27/2020 | Gamescom | 50 - 100 |
| 08/28/2020 | Liam Payne | 50 - 100 |
| 08/29/2020 | Steve Cohen | 50 - 100 |
| 08/30/2020 | Jon Rahm | 50 - 100 |
| 08/31/2020 | Payroll tax deferral | 50 - 100 |
| 09/01/2020 | Chris Paul | 50 - 100 |
| 09/02/2020 | Novichok | 50 - 100 |
| 09/03/2020 | Raised by Wolves | 100 - 200 |
| 09/04/2020 | SZA | 50 - 100 |
| 09/05/2020 | Shaquem Griffin | 50 - 100 |
| 09/06/2020 | Naomi Osaka | 50 - 100 |
| 09/07/2020 | Air quality index | 100 - 200 |
| 09/08/2020 | Kawhi Leonard | 100 - 200 |
| 09/09/2020 | The Walking Dead | 100 - 200 |
| 09/10/2020 | Tampa Bay Lightning | 100 - 200 |
| 09/11/2020 | Dallas Stars | 50 - 100 |
| 09/12/2020 | Iowa State football | 100 - 200 |
| 09/13/2020 | Grandparents Day | 200- 500 |
| 09/14/2020 | Snake | 200- 500 |
| 09/15/2020 | Blue Jays vs Yankees | 50 - 100 |
| 09/16/2020 | Final Fantasy 16 | 100 - 200 |
| 09/17/2020 | Monster Hunter Rise | 100 - 200 |
| 09/18/2020 | Yankees vs Red Sox | 50 - 100 |
| 09/19/2020 | UCF football | 50 - 100 |
| 09/20/2020 | Bad Bunny concert | 200- 500 |
| 09/21/2020 | Teddi Mellencamp | 50 - 100 |
| 09/22/2020 | Voter registration | 50 - 100 |
| 09/23/2020 | Moneybagg Yo | 50 - 100 |
| 09/24/2020 | RTX 3090 | 50 - 100 |
| 09/25/2020 | Lily Collins | 50 - 100 |
| 09/26/2020 | Baylor football | 50 - 100 |
| 09/27/2020 | Bam Adebayo | 200- 500 |
| 09/28/2020 | David Attenborough | 50 - 100 |
| 09/29/2020 | Steve Scully | 100 - 200 |
| 09/30/2020 | Iman Vellani | 100 - 200 |
| 10/01/2020 | Julio Jones | 100 - 200 |
| 10/02/2020 | Cookie Monsta | 100 - 200 |
| 10/03/2020 | Amanda Bynes | 50 - 100 |
| 10/04/2020 | Fleetwood Mac | 100 - 200 |
| 10/05/2020 | Rebel Wilson | 50 - 100 |
| 10/06/2020 | Mars | 50 - 100 |
| 10/07/2020 | Mexico | 50 - 100 |
| 10/08/2020 | Argentina | 50 - 100 |
| 10/09/2020 | Nobel Peace Prize | 50 - 100 |
| 10/10/2020 | Mental Health Day | 50 - 100 |
| 10/11/2020 | Cleopatra | 100 - 200 |
| 10/12/2020 | Paul Milgrom | 50 - 100 |
| 10/13/2020 | Oculus Quest 2 | 50 - 100 |
| 10/14/2020 | Tesla Model S | 50 - 100 |
| 10/15/2020 | Nikki Bella | 50 - 100 |
| 10/16/2020 | Mario Kart Live | 50 - 100 |
| 10/17/2020 | Jacinda Ardern | 50 - 100 |
| 10/18/2020 | Supermarket Sweep | 50 - 100 |
| 10/19/2020 | Katherine Schwarzenegger | 50 - 100 |
| 10/20/2020 | UEFA Champions League | 50 - 100 |
| 10/21/2020 | Blake Snell | 100 - 200 |
| 10/22/2020 | Europa League | 100 - 200 |
