# Supplementary material for: Using the COVID-19 Pandemic to Assess the Influence of News Affect on Online Mental Health-Related Search Behavior Across the United States: Integrated Sentiment Analysis and the Circumplex Model of Affect
Source: J Med Internet Res. 2022 Jan 27;24(1):e32731. doi: 10.2196/32731 (PMC8805454; doi:10.2196/32731)
Supplement: Multimedia Appendix 3 [file jmir_v24i1e32731_app3.doc]

Supplementary Table S3

*Additional Stopwords Used in News Story Title Preprocessing*

| anchorage | east | mass | port | third |
| --- | --- | --- | --- | --- |
| area | eastern | may | positive | three |
| associated | email | mayor | president | times |
| auburn | feel | mitt | press | today |
| back | first | month | print | town |
| baton | five | negative | province | tribune |
| bay | ford | net | record | trump |
| beach | forest | new | region | twin |
| brook | four | news | rock | two |
| brown | get | north | rouge | valley |
| cascade | glacier | northern | salt | video |
| cedar | globe | novel | say | virus |
| central | governor | ocean | says | watch |
| city | grand | one | sec | way |
| click | great | open | second | week |
| com | green | opens | see | west |
| comment | island | orange | share | western |
| could | king | palm | south | white |
| country | know | park | southern | window |
| county | lake | peninsula | state | wolf |
| dame | like | people | stony | would |
| day | little | phoenix | subscribe | year |
| door | live | place | tech |  |
