# Supplementary figures and images for: Using the COVID-19 Pandemic to Assess the Influence of News Affect on Online Mental Health-Related Search Behavior Across the United States: Integrated Sentiment Analysis and the Circumplex Model of Affect
Source: J Med Internet Res. 2022 Jan 27;24(1):e32731. doi: 10.2196/32731 (PMC8805454; doi:10.2196/32731)

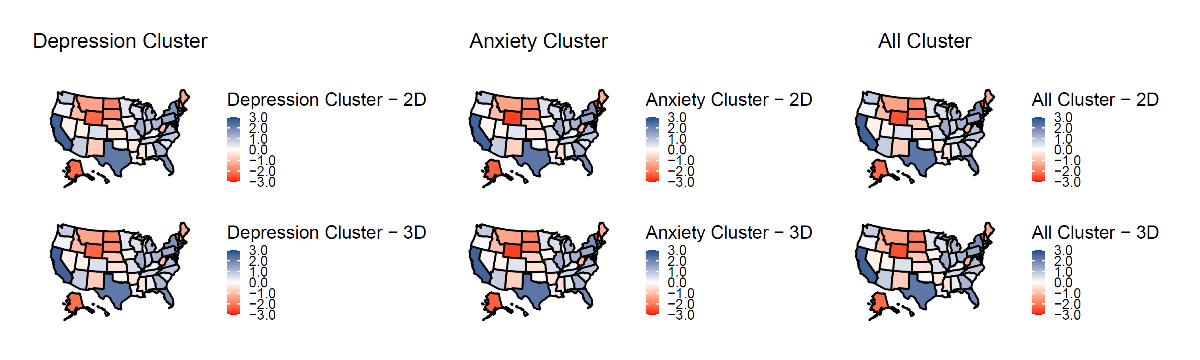

Supplement: Multimedia Appendix 6 [file jmir_v24i1e32731_app6.png]
